# Qi-Li-Qiang-Xin Alleviates Isoproterenol-Induced Myocardial Injury by Inhibiting Excessive Autophagy *via* Activating AKT/mTOR Pathway

**DOI:** 10.3389/fphar.2019.01329

**Published:** 2019-11-12

**Authors:** Cailian Fan, Xiyang Tang, Mengnan Ye, Guonian Zhu, Yi Dai, Zhihong Yao, Xinsheng Yao

**Affiliations:** ^1^College of Traditional Chinese Materia Medica, Shenyang Pharmaceutical University, Shenyang, China; ^2^College of Pharmacy and International Cooperative Laboratory of Traditional Chinese Medicine Modernization and Innovative Drug Development of Chinese Ministry of Education, Jinan University, Guangzhou, China; ^3^Research Core Facility, West China Hospital, Sichuan University, Chengdu, China

**Keywords:** Qi-Li-Qiang-Xin, myocardial injury, isoproterenol, apoptosis, autophagy

## Abstract

**Background:** Apoptosis and autophagy are two important patterns of cell death in the process of heart failure. Qi-Li-Qiang-Xin (QLQX), a traditional Chinese medicine, has been frequently used in the treatment of chronic heart failure (CHF) in China. However, the potential effect of QLQX on autophagy has not been reported. In this study, we aimed to investigate whether QLQX alleviated isoproterenol (ISO)-induced myocardial injury through regulating autophagy.

**Methods:** The rapid identification of chemical ingredients of QLQX was performed by UPLC-Q-TOF-MS, and the contents of major constituents in QLQX were also measured by UPLC-Q-TOF-MS. ISO was used to induce myocardial injury in H9c2 cardiomyocytes and SD rats. *In vivo*, cardiac function was evaluated by echocardiography and cardiac structure was observed by HE and Masson staining. Expressions of Bcl-2, Bax, LC3, P62, AKT, p-AKT, mTOR, and p-mTOR were detected by western blotting. *In vitro*, H9c2 cells were pretreated with QLQX for 3 h before ISO (80 µM, 48h) addressed. Cell viability, LDH and CK-MB release, apoptosis ratio, and the level of autophagy were measured. Western blotting was also performed to detected related protein expressions.

**Result:** *In vivo*, treatment by QLQX significantly improved cardiac function and alleviated ISO-induced myocardial structural damage. In addition, QLQX markedly decreased apoptosis and inhibited autophagic activity, accompanied by activating the AKT/mTOR pathway. *In vitro*, the increased cell apoptosis induced by ISO was paralleling with the gradually increasing level of autophagy. Furthermore, 3-MA, an autophagic inhibitor, could block ISO-induced autophagy in H9c2 cells. Our results suggested that both QLQX and 3-MA treatment could decrease cell death induced by ISO, implying that QLQX protected against ISO-induced myocardial injury possibly by inhibiting excessive autophagy-mediated cell death. In addition, blockage of AKT signaling by an AKT inhibitor, capivasertib, could reduce the effect of QLQX on inhibiting ISO-induced apoptosis and autophagy-mediated cell death.

**Conclusion:** QLQX could alleviate ISO-induced myocardial injury by inhibiting apoptosis and excessive autophagy-mediated cell death *via* activating the AKT/mTOR pathway.

## Introduction

Chronic heart failure (CHF) is a potentially fatal physiological condition in which cardiac output is unable to satisfy the needs of the body (Doehner and Anker, 2010). It can be caused by a variety of etiologic factors, including myocardial infarction, hypertension, amyloidosis, and so on (John and Piotr, 2011). The exact evidence has indicated that concentrations of catecholamines are elevated in the development of heart failure, resulting in morphological alterations of the heart or left ventricular hypertrophy (Bristow et al., 1985; Lefkowitz et al., 2000). Isoproterenol (ISO), one of the catecholamine adrenergic receptor agonists, has been proved to cause myocardial necrosis, cardiac hypertrophy, fibroblast proliferation, and abnormality of diastolic and systolic functions by subcutaneous injection, and these changes of heart induced by ISO can mimic the pathological changes of cardiac tissue in the process of human heart failure (Benjamin et al., 1989; Oudit et al., 2003; Christian et al., 2005; Wang et al., 2018). Therefore, the model of ISO-induced myocardial injury has been widely used in exploring the beneficial effect of drugs on cardiac dysfunction.

In the past few years, there has been a growing awareness that autophagy plays a homeostatic role in cardiac development under physiological conditions (Lampert and Gustafsson, 2018). Autophagy is a lysosomal degradation pathway that involves the delivery of cytoplasmic cargo to the lysosome organelles (Beth and Guido, 2008). Piles of evidences have reflected that autophagy plays a dual role in myocardial injury. At the basal level, almost all cells undergo autophagy to maintain energy metabolism and substance reuse (Kourtis and Tavernarakis, 2009). And it is rapidly upregulated when cellular energy cannot meet by nutritional supply, such as during starvation and hypoxia (Kourtis and Tavernarakis, 2009). Therefore, enhancing autophagy at a moderate level could protect against hemodynamic or neurohormonal stresses in the process of heart failure (Kourtis and Tavernarakis, 2009; Zilinyi et al., 2018). However, excessive activation of autophagy leads to excessive self-digestion and degradation of essential cellular constituents, which could cause autophagic cell death (Gustafsson and Gottlieb, 2009; Sciarretta et al., 2018; Wang et al., 2018). Zhu et al. found that cardiac autophagy triggered by chronic load was an inadaptable response that contributed to the progression of heart failure (Zhu et al., 2007). Thus, prevention of excessive autophagy maybe a promising strategy to improve cardiac dysfunction.

Qi-Li-Qiang-Xin (QLQX) is a traditional Chinese medicine (TCM) prescription for the prevention and treatment of CHF. It is composed of 11 herbal medicines, including Astragali Radix [the roots of *Astragalus membranaceus* (Fisch.) Bunge], Aconiti Lateralis Radix Preparata (the processed daughter roots of *Aconitum carmichaelii* Debeaux), Ginseng Radix et Rhizoma (the roots and rhizomes of *Panax ginseng* C.A.Mey.), Salvia Miltiorrhiza Radix et Rhizoma (the roots and rhizomes of *Salvia miltiorrhiza* Bunge), Alismatis Rhizoma [the rhizomes of *Alisma plantago-aquatica* subsp. *orientale* (Sam.) Sam.], Descuraunia Semen [the seeds of *Descurainia sophia* (L.) Webb ex Prantl], Cinnamomi Ramulus [the dried barks of *Cinnamomum cassia* (L.) J. Presl], Carthami Flos (the dried flowers of *Carthamus tinctorius* L.), Periplocae Cortex (the root barks of *Periploca sepium* Bunge), Polygonati Odorati Rhizoma [the dried rhizomes of *Polygonatum odoratum* (Mill.) Druce], and Citri Reticulatae Pericarpium (the firut peels of *Citrus reticulata* Blanco) (Sun et al., 2016). Among them, Astragali Radix and Aconiti Lateralis Radix Preparata are the monarch medicines (*Jun*) in the prescription. According to the TCM theory, CHF is considered as blood stasis due to *Qi* deficiency and water overflowing due to *Yang* deficiency. Both of these two monarch medicines could warm *Yang* to promote diuresis and supplement *Qi* to remove blood stasis. Salvia Miltiorrhiza Radix et Rhizome, Descuraunia Semen, and Ginseng Radix et Rhizome are selected as the minister medicines (*Chen*). Salvia Miltiorrhiza Radix et Rhizoma could nourish blood and promote blood circulation. Descuraunia Semen is used for purging lung and diuresis, and Ginseng Radix et Rhizoma could invigorate *Qi*. In addition, Carthami Flos, Periplocae Cortex, Alismatis Rhizome, and Polygonati Odorati Rhizoma are adjuvant medicines (*Zuo*). Carthami Flos could promote blood circulation. Periplocae Cortex, Alismatis Rhizome, and Polygonati Odorati Rhizoma are benefit on diuresis and detumescence. Cinnamomi Ramulus and Citri Reticulatae Pericarpium are used as guiding medicines (*Shi*). Both of them could warm *Yang* and transform *Qi* to increase the efficacy of Astragali Radix and Aconiti Lateralis Radix Preparata. Besides, the efficacy of QLQX on the treatment of CHF has been confirmed by a randomized clinical trial (Li et al., 2013). Our previous study on chemical recognition of QLQX by UPLC/Q-TOF-MS has showed that it contains saponins and flavonoids (mostly originated from Astragali Radix and Ginseng Radix et Rhizoma), diterpene alkaloids (originated from Aconiti Lateralis Radix Preparata), cardiac glycosides (mainly originated from Periplocae Cortex), phenolic acids, and diterpene quinones (originated from Salvia Miltiorrhiza Radix et Rhizome and Descuraunia Semen) (Yun et al., 2018). Studies have suggested that QLQX plays the role in protecting myocardial injury through regulating the inflammatory, oxidative stress, and energy metabolism (Xiao et al., 2009; Zhang et al., 2013). However, there is no study to explore the effect of QLQX on autophagy in the treatment of heart failure. Therefore, we aimed to investigate the function of QLQX in regulating autophagy exposed to ISO-induced CHF and identified the potential molecular mechanisms involved.

## Materials and Methods

### Materials

QLQX(A20170820) was obtained from Shijiazhuang Yiling Pharmaceutic (Hebei, China). Metoprolol tartrate was purchased from AstraZeneca Pharma (Jiangsu, China). Isoprenaline Hydrochloride, 3-MA and 3-[4,5dimethylthiazol-2-yl]-2,5 diphenyl tetrazolium (MTT) were purchased from Sigma (St. Louis, MO, USA). Capivasertib was obtained from MCE (USA). The Dulbecco’s modified Eagle’s medium (DMEM) and the fetal bovine serum (FBS) were purchased from Hyclone (Logan, USA). The antibodies against GAPDH (#5174), Bcl-2 (#3498), Bax (#2774), P62 (#16177), AKT (#9272) and p-AKT (#4060), mTOR (#2972) and p-mTOR (#5536), Anti-mouse IgG-HRP-linked Antibody (#7076), and Anti-rabbit IgG-HRP-linked Antibody (#7074) were purchased from Cell Signaling Technology (Danvers, USA). The antibody against LC3 (ab51520) was obtained from Abcam (Cambridge, MA, USA). ECL detection system was purchased from Santa Cruz Biotechnology (Santa Cruz, USA).

### Preparation of Test Drugs

The powder of QLQX was dissolved in methanol. After 30 min of ultrasonic dissolution, the solution was centrifuged at 1,4000 r·min^–1^ for 10 min. Then, 2 µl of the supernatant was prepared to analyze. According to the method of Yun et al. (2018), the rapid identification of 152 chemical ingredients of QLQX was performed by UPLC-Q-TOF-MS (**Figure S1** and **Table S1**). As sinapine thiocyanate, calycosin-7-O-β-D-glucopyranoside, hesperidin, salvianolic acid B, benzoylmesaconine, ginsenoside Re, periplocin, ginsenoside Rb1, formononetin, periplocymarin, astragalosides II, and alisol A were the predominant exposure components in plasma after oral administration of QLQX in rats, they were considered as representative quality control markers for QLQX. The chemical structures of these compounds were provided in **Figure S2**, and their concentrations in QLQX were also determined as follows: sinapine thiocyanate 0.79 mg/g, calycosin-7-O-β-D-glucopyranoside 0.25 mg/g, hesperidin 0.20 mg/g, salvianolic acid B 3.13mg/g, benzoylmesaconine 0.10 mg/g, ginsenoside Re 0.43 mg/g, periplocin 0.31 mg/g, ginsenoside Rb1 1.15 mg/g, formononetin 0.08 mg/g, periplocymarin 0.27 mg/g, astragalosides II 0.51 mg/g, and alisol A 0.62 mg/g (**Table S2**).

### Experimental Animals and Animal Models of CHF

Male Sprague Dawley (SD) rats were provided by Hua Fukang Biotechnology Company (Beijing, China). The rats (weight 150–200 g) were housed with five each in sanitized polypropylene cages at room temperature for 12-h light/dark cycle. This study was carried out in accordance with the principle of the Basal Declaration and Recommendations of Laboratory Animal Ethics Committee of West China Hospital, Sichuan University. The protocol was approved by Laboratory Animal Ethics Committee of West China Hospital, Sichuan University (A2019118A). ISO was abdominally and subcutaneously injected for 7 days at a dose of 5 mg/kg body weight to build the animal model of CHF (Kralova et al., 2008; Zhou et al., 2017). LVEF < 55% by echocardiography was defined as CHF. The control group (n = 8) was fed a dose of saline (6 ml/kg/day) for 4 weeks, and rats that were identified to be in the state of heart failure were randomly divided into five groups: ISO group, different doses of QLQX-treated groups (0.3, 0.6, 1.2 g/kg, *i.g*., every day) and metoprolol group (10 mg/kg, *i.g*., every day). Rats were anesthetized by inhaling 3% isoflurane when they were measured cardiac function by ultrasound M-mode echocardiography.

### Echocardiography

The M-mode echocardiogram system (Vivid i; GE, America) with a 13-MHz ultrasonic probe (12L-RS Linear Probe; GE, America) was used to evaluate the cardiac function of rats. The echocardiogram was recorded in the left ventricle short axis to measure the left ventricular ejection fraction (LVEF) and fractional shortening (FS). Five uninterrupted cardiac cycles were obtained from each rat.

### Histopathological Study

The left ventricles of rats were immersed in 4% paraformaldehyde for 48 h and then embedded in paraffin as previously described (Sun et al., 2018). Then, 4-µm thick serial sections were obtained, deparaffinized, and rehydrated. The sections of left ventricles were stained with Masson’s trichrome and hematoxylin and eosin (HE). Pathological examination was performed under light microscopy (Axio Imager A2, Zeiss, Germany) for observation of structural abnormality.

### Terminal Deoxynucleotidyl Transferase Dutp Nick-End Labeling (TUNEL) Staining

The apoptotic in cardiac tissue was evaluated by *In Situ* Cell Death Detection Kit according to the manufacturer’s instructions. Briefly, the left ventricles of rats were fixed in 4% paraformaldehyde for 48 h and then embedded in paraffin. Then, 4-µm thick serial sections were made, deparaffinized, dehydrated in graded alcohol, and stained by the regents in the *In Situ* Cell Death Detection Kit. The apoptotic nuclei stained by TUNEL were brownish yellow. All these figures were analyzed by Image-Pro Plus 6.0 (Media Cybernetics, USA).

### Immunohistochemistry of Cardiac Tissues

Immunohistochemistry analysis of left ventricular was used to evaluate the level of autophagy. In brief, sections of 4 µm in thickness were prepared. After antigen retrieval, sections were incubated with 0.3% hydrogen peroxide at room temperature for 25 min, then blocked in 5% BSA in PBS, and then incubated with rabbit antibodies against LC3 (1:300) at 4°C overnight. After that, an anti-rabbit secondary antibody was used to incubate sections at room temperature for 120 min. The developed tissue sections were imaged with an upright metallurgical microscope (Axio Imager A2, Zeiss, Germany). All these figures were analyzed by Image-Pro Plus 6.0 (Media Cybernetics, USA).

### Cell Culture

H9c2 cell line (rat embryonic ventricular myocytes) were purchased from the Shanghai Institute of Biochemistry and Cell Biology (China). Cells were cultured in high-glucose DMEM medium supplemented with 100 U/ml penicillin, 100 µg/ml streptomycin, and 10% FBS at the condition of 37°C and 5% CO_2_. When the cells were grown to a density of 80%–90% in culture medium, cell passage was done. The medium was changed 2–3 times per week.

The ISO model of H9c2 cells was built to mimic myocardial injury *in vivo*. Briefly, when H9c2 cells have grown to 80% confluence, the culture medium was removed and, H9c2 cells were washed third times with 1% FBS DMEM. The cells were pretreated with QLQX at different concentrations for 3 h and then exposed to isoproterenol (80 µM) for 48 h using 1% FBS DMEM.

### Detection of Cell Viability

H9c2 cells were cultured at a density of 6 × 10^4^ cells/ml in 96-well plates. Cells were pretreated with QLQX for 3 h and then incubated with ISO (80 µM) for 48 h using 1% FBS DMEM. After that, the medium was replaced by FBS-free DMEM with 20 µl of 5 mg/ml MTT for 4 h. Then, the supernatant was removed, and the formazan was dissolved in 150 µl of DMSO with a shaker for 3 min. The optical density (OD) was measured at a 490 nm wavelength using a Multi-Reader instrument (Biotek, BioTek Instruments Inc., USA).

### Determination of Apoptosis Ratio

Apoptosis was measured using the Annexin-V-FLUOS Staining Kit (Roche, Germany) according to the manufacturer’s protocol. Briefly, H9c2 cells were treated with ISO and QLQX for 48 h. After harvested, cells were stained with the Annexin-V-FLUOS staining reagents for 15 min. The apoptotic cells were quantified using a flow cytometer (Beckman Coulter). The apoptosis rate was reflected *via* calculating the ratio of AnnexinV-positive/PI- positive cells to total cells.

### Detection of Cellular Autophagy

Cyto-ID® Autophagy Detection Kit (Enzo Life Sciences, Farmingdale, NY, USA) was used for detection of cellular autophagy. According to the manufacturer’s instruction, H9c2 cells were harvested, then washed with cold PBS twice and incubated with Cyto-ID® Green staining solution for 30 min in the dark. Then, cells were checked on a CytoFLEX flow cytometer (Beckman Coulter). Three independent experiments were performed.

### Western Blot Analysis

The expression levels of Bcl-2, Bax, LC3, P62, AKT, p-AKT, mTOR, p-mTOR proteins in left ventricular tissues and H9c2 cells were evaluated using western blot analysis. Approximately, 100 mg of heart tissues were homogenized by using a polytron homogenizer in RIPA buffer with protein phosphatase inhibitor. And H9c2 cells were harvested and digested by the same way. After the lysates were harvested, protein concentrations of samples were measured using a bovine serum albumin kit (Bio-Rad Laboratories, Hercules, USA). Analysis of western blot was carried out as follows: equal amounts of protein were boiled for 10 min and separated by SDS-PAGE on 8%–12% gels. The proteins were transferred to membranes and then incubated with primary antibodies Bcl-2 (1:1,000), Bax (1:1,000), LC3 (1:2,000), P62 (1:1,000), AKT (1:1,000), p-AKT (1:1,000), mTOR (1:1,000), p-mTOR (1:1,000), and GAPDH (1:3,000) at 4°C overnight. Then, the membranes were incubated for 2 h with secondary antibodies (1:4,000) at room temperature. Immunoreactive bands were visualized by using ECL as the HRP substrate. The protein signals of protein bands in membranes were then captured using Image Lab™ Software (Bio-Rad, Shanghai, China) and quantified using Image J software program.

### Statistical Analysis

All statistical analyses were calculated by one-way analysis of variance (ANOVA) followed by Scheffe’s *post hoc* test. Data were collected from repeated experiments and presented as mean ± SD. *p* < 0.05 was considered to have a statistical difference.

## Results

### QLQX Significantly Improves Cardiac Function in Rats Subjected to ISO-Induced Injury

**Figure 1:**
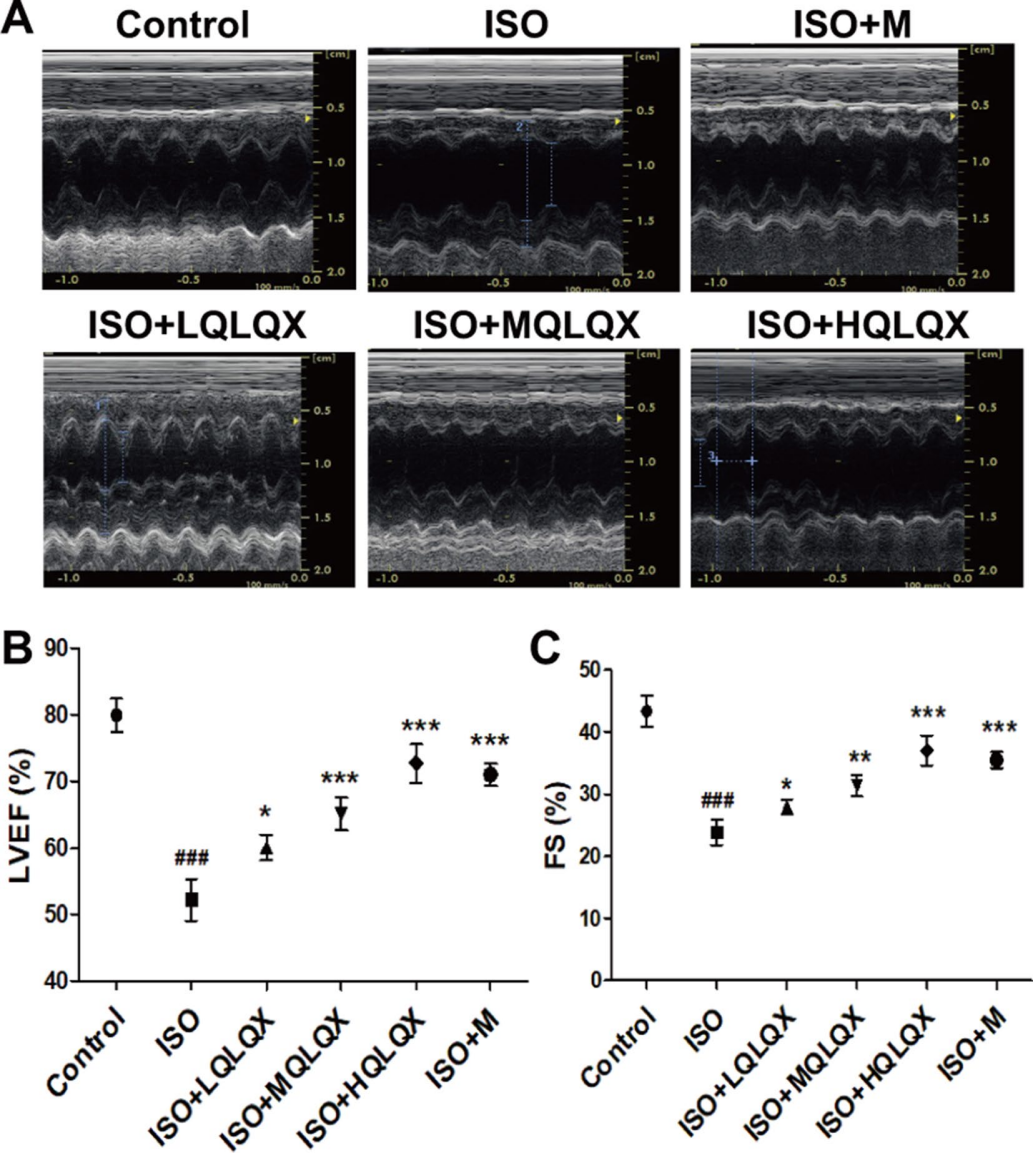
QLQX treatment ameliorates cardiac function in the rats of CHF induced by ISO. **(A)** Representative M mode images of echocardiography for the different groups. **(B**, **C)** Quantitative analysis of cardiac function by echocardiography measurement: LVEF **(B)**, FS **(C)**. Data are expressed as mean ± SD, n = 8. ^###^ *p* < 0.001 *versus* control group; *p < 0.05, **p < 0.01, ***p < 0.001 *versus* ISO group. CHF, chronic heart failure; LVEF, left ventricular ejection fraction; QLQX, Qi-Li-Qiang-Xin, ISO, isoproterenol.

To uncover the effect of QLQX on ISO-induced CHF rats, cardiac indices such as LVEF, FS were measured by echocardiography. As shown in **Figures 1A, B**, LVEF of the ISO group showed a lower level than the control group, but LVEF of the ISO+MQLQX and ISO+HQLQX group was significantly increased compared with the ISO group (*p* < 0.01). Meanwhile, MQLQX and HQLQX treatment markedly increased FS by comparison of the ISO group (*p* < 0.05, **Figures 1A, C**). Notably, the ameliorative effect of HQLQX in CHF rats was comparable to the effect of positive control metoprolol (*p* > 0.05). In this study, we also showed that chronic stimulation with ISO could induce hypertrophic hearts evidenced by marked increase of HW/BW ratio. After treatment by QLQX four consecutive weeks, the ratio of HW/BW was significantly decreased in CHF rats (*p* < 0.01, **Figure S3**).

### QLQX Ameliorates Myocardial Tissue Structure and Decreases the Myocardial Fibrosis in CHF Rats

**Figure 2:**
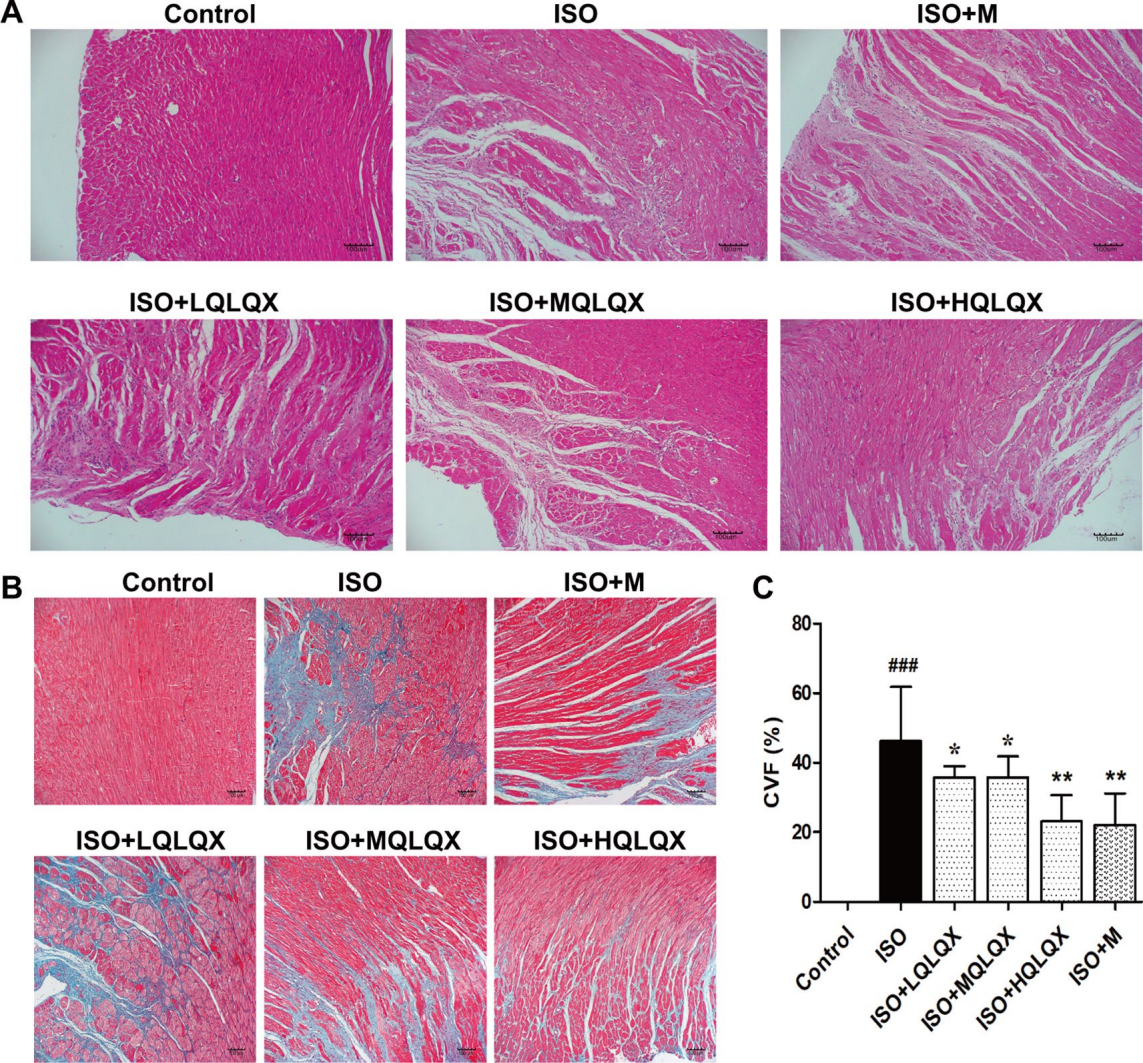
QLQX improves myocardial tissue structure and reduces cardiac fibrosis in the rats of CHF. **(A)** Representative HE staining images of left ventricles (×100) for the different groups treated by ISO with or without QLQX. **(B)** Representative images of Masson staining (×100) for the different groups treated by ISO with or without QLQX. **(C)** Quantitative analysis of collagen volume of left ventricle. Data are expressed as mean ± SD, n = 3. ^###^ *p* < 0.001 *versus* control group; *p < 0.05, ^**^ *p* < 0.01 *versus* ISO group. ISO, isoproterenol.

To evaluate the protective effect of QLQX on myocardial tissue structure, HE staining was used. It was apparent from **Figure 2A** that the cardiomyocytes in the control group showed an orderly arrangement, and the nuclei of cardiomyocytes were in the center of the muscle fibers neatly. Contrary to the control group, severe cardiomyocyte necrosis and nuclear dissolution were observed in the ISO group, while myocardial structures were largely maintained in the ISO+MQLQX, ISO+HQLQX and positive control group.

The myocardial fibrosis of the hearts was evaluated by Masson’s trichrome staining. **Figures 2B, C** directly displayed that a bigger area of myocardial fibrosis in the ISO group was observed, while ISO+MQLQX, ISO+HQLQX and positive control group drastically reduced the area of myocardial fibrosis compared with the ISO group (*p* < 0.01). These results suggested that QLQX could improve myocardial tissue structure and decrease the myocardial fibrosis in ISO-induced CHF rats.

### QLQX Inhibits ISO-Induced Myocardial Apoptosis and Excessive Autophagy Related to the AKT/mTOR Pathway *In Vivo*

**Figure 3:**
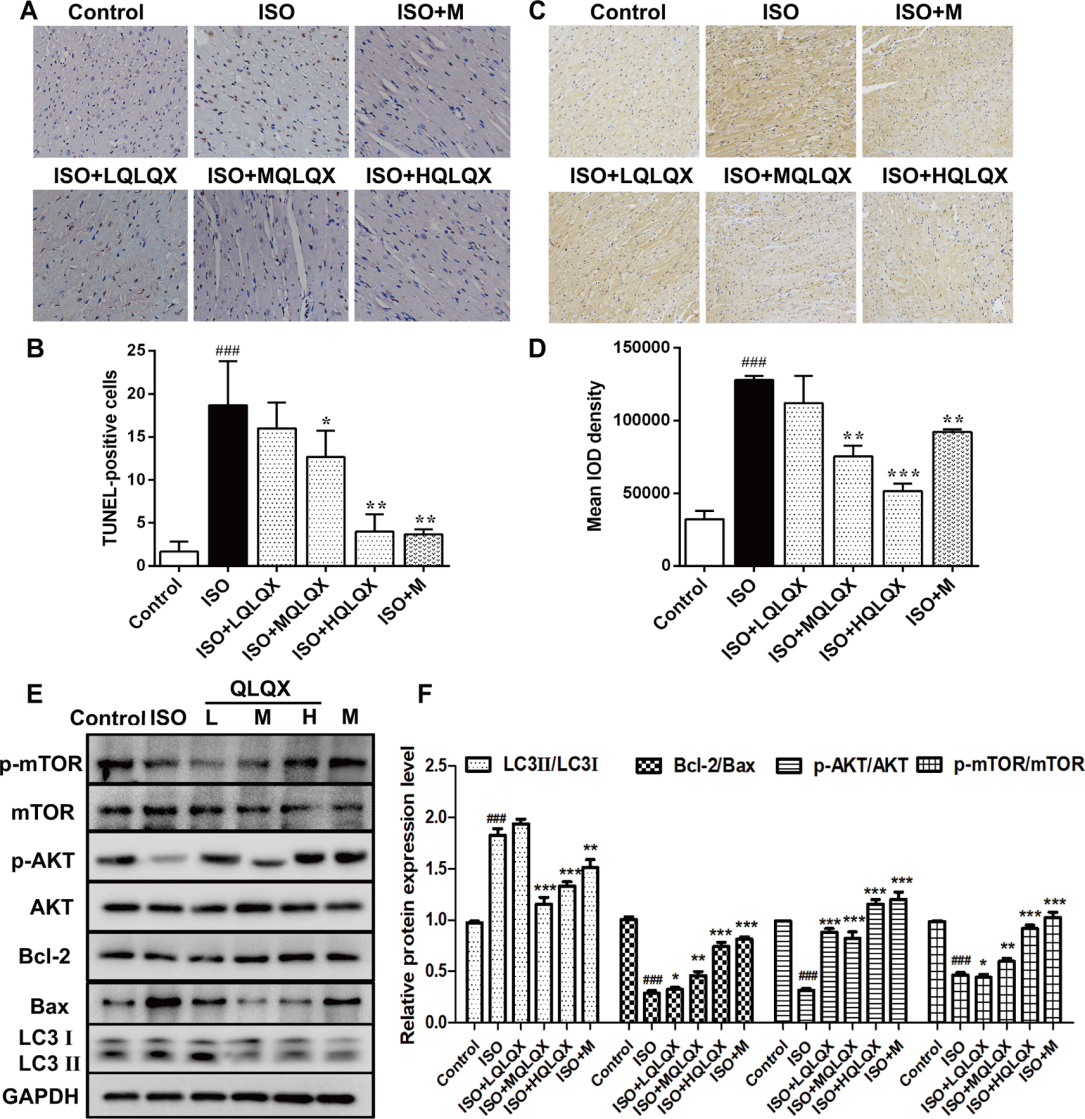
QLQX inhibits ISO-induced myocardial apoptosis and autophagy and involves in regulating the AKT/mTOR pathway *in vivo*. **(A)** Representative images of TUNEL staining (×400) for the different groups treated by ISO with or without QLQX. **(B)** Quantitative analysis of the proportion of TUNEL-positive cells. **(C)** Representative IHC images of LC3 in rats left ventricles (×100) for the different groups treated by ISO with or without QLQX. **(D)** Quantitative analysis of integrated optical density of LC3. **(E)** Representative western blotting bands of LC3I, LC3II, Bcl-2, Bax, p-AKT, AKT, p-mTOR, and mTOR and quantitative analysis of the ratios of LC3II/LC3I, Bcl-2/Bax, p-AKT/AKT, p-mTOR/mTOR by densitometry based on immunoblot images. Data are expressed as mean ± SD, n = 3. ^###^ *p* < 0.001 *versus* control group; *p < 0.05, **p < 0.01, ***p < 0.001 *versus* ISO group. ISO, isoproterenol; QLQX, Qi-Li-Qiang-Xin.

To investigate the effect of QLQX on ISO-induced apoptosis *in vivo*, we examined apoptosis on cardiac tissues by TUNEL staining. As showed in **Figures 3A, B**, the ISO group had more TUNEL-stained cells than the control group (*p* < 0.01), while MQLQX and HQLQX treatment notably reduced the number of TUNEL-stained cells compared with the ISO group (*p* < 0.01). Then, we checked the level of autophagy using immunohistochemistry. As showed in **Figures 3C, D**, compared with the control group, LC3 level was increased in the ISO group (*p* < 0.001), while MQLQX and HQLQX treatment significantly decreased the level of LC3 (*p* < 0.01). Besides, expressions of apoptosis and autophagy related proteins were examined by western blotting (**Figure 3E**). The ISO group showed a marked decrease in the ratio of Bcl-2/Bax and increase in the ratio of LC3II/LC3I (*p* < 0.001), while MQLQX and HQLQX treatment made a significant increase of Bcl-2/Bax ratio and reduction of LC3II/LC3I ratio (*p* < 0.01, **Figure 3E**). From these data, we could find that ISO enhanced apoptosis and autophagy *in vivo*, while MQLQX and HQLQX could attenuate such abnormalities. The similar changes of related protein expressions in positive control metoprolol group were also observed. These results suggested that QLQX could inhibit ISO-induced myocardial apoptosis and excessive autophagy-mediated cell death.

Western blotting analysis also pointed out that the ratios of p-AKT/AKT and p-mTOR/mTOR in the ISO group were obviously decreased compared with the control group (*p* < 0.001, **Figures 3E, F**), while MQLQX and HQLQX treatment remarkably increased their expressions in rats (*p* < 0.001, **Figures 3E, F**). These results suggested that QLQX inhibited ISO-induced myocardial apoptosis and excessive autophagy, which was involved in activating the AKT/mTOR pathway.

### ISO Induces Cell Death and Autophagy in H9c2 Cells

**Figure 4:**
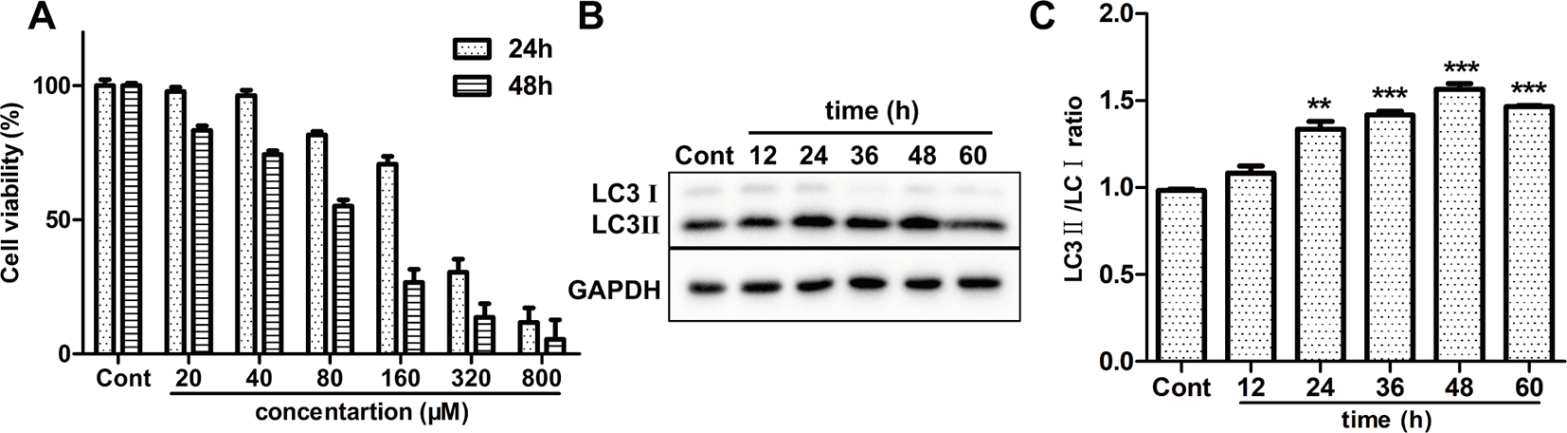
Effect of ISO on cell death and autophagy on H9c2 cells. **(A)** The viability of H9c2 cells was tested by MTT treated by different concentrations of ISO. **(B)** Representative western blotting bands of LC3I, LC3II in H9c2 cells treated by ISO for 0, 12, 24, 36, 48, and 60 h. **(C)** Quantified the ratio of LC3II/LC3I by densitometry based on immunoblot images. Data are expressed as mean ± SD, n = 3. **p < 0.01, ***p < 0.001 *versus* ISO group. ISO, isoproterenol.

To examine the cytotoxic effect of ISO on H9c2 cardiomyocytes, MTT method was used. A range of concentrations of ISO were used to conform the effective concentration of ISO that could result in 40%–50% cell death. As shown in **Figure 4A**, treated by 80 µM ISO for 48h, H9c2 cells showed the survival ratio of 50%–60%. Then, 80 µM ISO for 48 h was selected to induce myocardial injury in H9c2 cells. Furthermore, we monitored the level of autophagy using western blotting treated by 80 µM ISO for 0, 12, 24, 48, and 72 h. As displayed in **Figures 4B, C**, the expressional ratio of LC3II/LC3I protein was upregulated over time (*p* < 0.001), while the expressional ratio of LC3II/LC3I protein had a decreasing trend after 48 h. These data suggested that ISO-induced cell death was related to the level of autophagy in H9c2 cells.

### QLQX Significantly Improves Cell Viability and Alleviates Myocardial Injury

**Figure 5:**
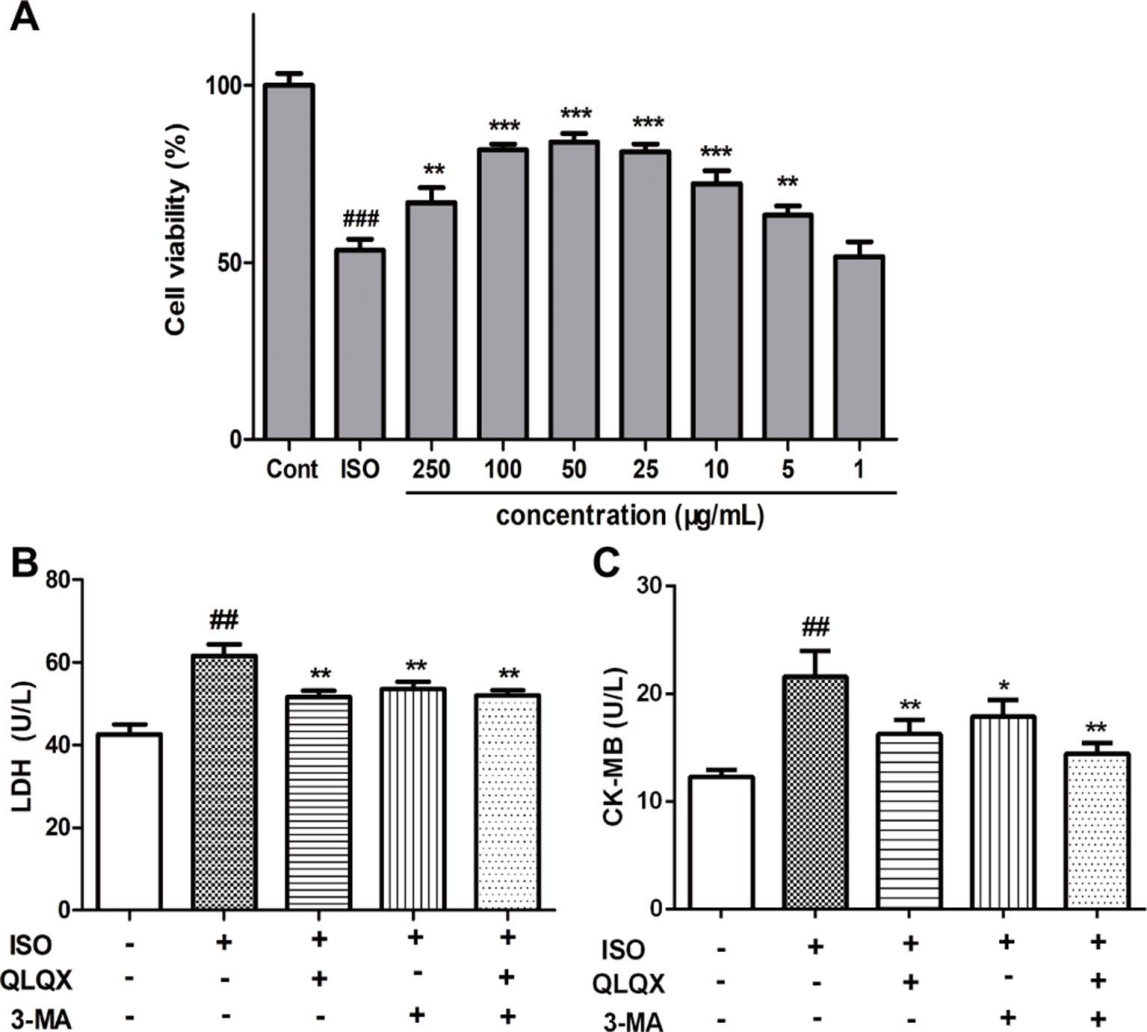
QLQX improves cell viability and alleviates ISO-induced myocardial injury in H9c2 cells, which is similar to the treatment by 3-MA. **(A)** The viability of H9c2 cells was tested by MTT in different concentrations of QLQX. **(B)** The release of LDH in the culture medium in different groups. **(C)** The activity of CK-MB in the culture medium in different groups. Data are expressed as mean ± SD, n = 3. ^##^ *p* < 0.01, ^###^ *p* < 0.001 *versus* control group; *p < 0.05, **p < 0.01, ^***^ *p* < 0.001 *versus* ISO group. QLQX, Qi-Li-Qiang-Xin, ISO, isoproterenol.

To investigate the cytotoxic effect of QLQX on H9c2 cells, H9c2 cells were treated with different concentrations of QLQX (1,000, 500, 250, 100, 50, 25, 5, and 1 µg/ml) for 48 h. While treatment with 1,000 µg/ml QLQX had a significant inhibition of cell viability (*p* < 0.01), treatment with QLQX (500, 250, 100, 50, 25, 5, and 1 µg/ml) for 48 h had little effect (*p* > 0.05, **Figure S4**). Then, a range of concentrations of QLQX (250, 100, 50, 25, 10, 5, and 1 µg/ml) were used to evaluate the protective effect of QLQX on ISO-induced injury *in vitro*. As exhibited in **Figure 5A**, the cell viability was higher in the QLQX (250, 100, 50, 25, and 5 µg/ml) group than the ISO group (*p* < 0.05). As the concentration of 50 µg/ml depicted the highest cell survival ratio, this concentration was selected for the next investigation.

LDH and CK-MB are diagnostic marker enzymes of myocardial injury. So, the release of LDH and CK-MB was measured. **Figures 5B, C** showed that the LDH leakage and CK-MB activity in the ISO group were greatly increased than the control group (*p* < 0.01), while 50 µg/ml QLQX pretreatment significantly reduced both of them compared with the ISO group (*p* < 0.01). Furthermore, 3-MA (3 mM), an autophagic inhibitor, was also displayed the similar effect to QLQX. Both of them could alleviate ISO-induced myocardial injury.

### QLQX Alleviates Cell Apoptosis Ratio and Suppresses ISO-Induced Excessive Autophagy

**Figure 6:**
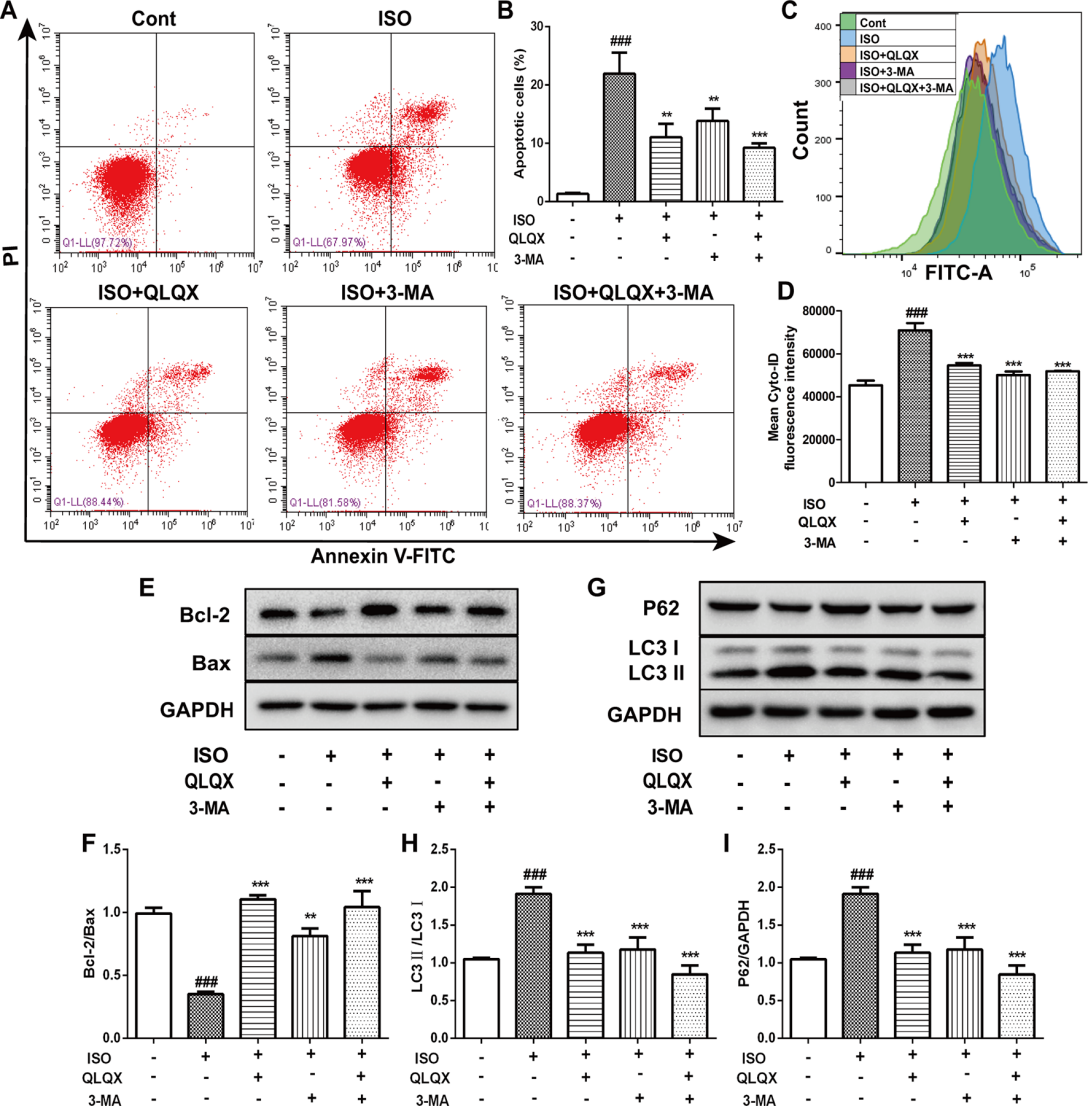
QLQX inhibits apoptosis and excessive autophagy induced by ISO in H9c2 cells. **(A)** Representative images of the total apoptotic cells by annexin V-FITC/PI detection. **(B)** Quantitative analysis of apoptotic cells using bar graphs. **(C)** Cellular autophagy measured by Cyto-ID autophagy detection reagent by flow cytometry. **(D)** Quantitative analysis of mean fluorescence intensity in different groups. **(E)** Representative western blotting bands of Bcl-2 and Bax. **(F)** Quantitative analysis of the ratio of Bcl-2/Bax by densitometry based on immunoblot images. **(G)** Representative western blotting bands of LC3I, LC3II and P62. **(H**, **I)** Quantitative analysis of the ratios of LC3II/LC3I and P62/GAPDH by densitometry based on immunoblot images. Data are expressed as mean ± SD, n = 3. ^###^ *p* 0.001 *versus* control group; **p < 0.01, ***p < 0.001 *versus* ISO group. ISO, isoproterenol; QLQX, Qi-Li-Qiang-Xin.

To explore the effect of QLQX on ISO-induced apoptosis, we monitored the number of apoptotic cells by flow cytometry. The number of apoptotic cells was greatly increased in the ISO group (*p* < 0.01), while QLQX and 3-MA treatment markedly decreased cell apoptosis (*p* < 0.01, **Figures 6A, B**). In addition, apoptosis-related proteins Bcl-2 and Bax were measured by western blotting. As shown in **Figures 6E, F**, QLQX and 3-MA could obviously increase the ratio of Bcl-2/Bax compared with the ISO group (*p* < 0.01). All these results clearly indicated that QLQX and 3-MA could effectively inhibit ISO-induced apoptosis.

The level of autophagy was measured through labeling autophagic compartment by Cyto-ID staining using flow cytometry. ISO induced a marked augment of fluorescence intensity of Cyto-ID (*p* < 0.001), while QLQX and 3-MA treatment dramatically reduced fluorescence intensity (*p* < 0.001, **Figures 6C, D**). Additionally, the expressions of autophagy-related proteins LC3 and P62 were also examined. ISO induced an increase in the conversion of LC3I to LC3II, whereas the ratio of LC3II/LC3I was significantly down-regulated by QLQX and 3-MA (*p* < 0.01, **Figures 6G, H**). However, the protein expression of P62 displayed the opposite change (**Figures 6G, I**). These results suggested that QLQX could inhibit ISO-induced apoptosis and autophagy.

### QLQX Inhibits Apoptosis and Autophagy Through the AKT/MTOR Pathway

**Figure 7:**
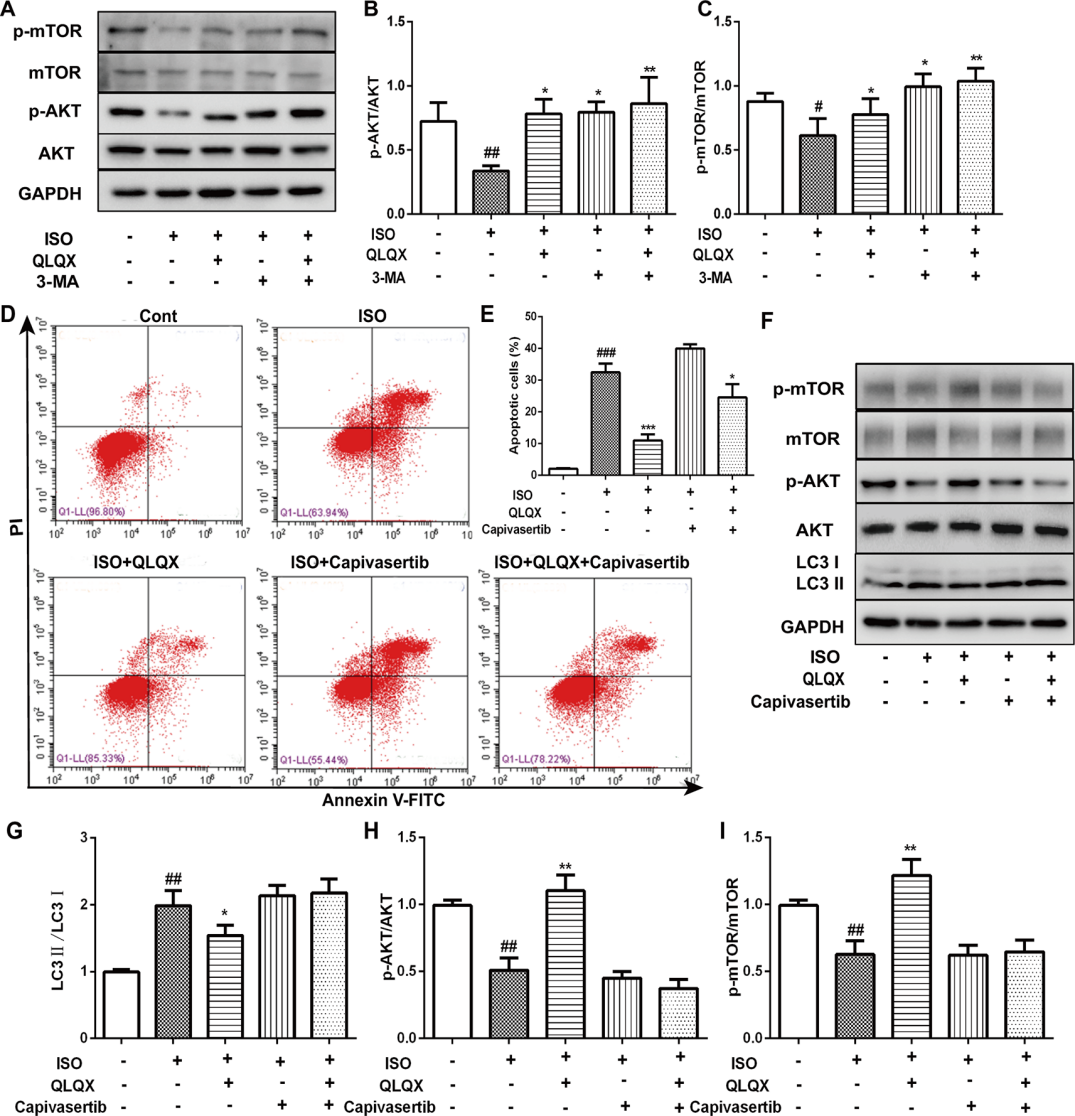
The role of AKT/mTOR pathway in cardioprotective effect of QLQX *in vitro*. **(B)** Representative western blotting bands of p-AKT, AKT, p-mTOR and mTOR. **(B**, **C)** Quantitative analysis of the ratios of p-AKT/AKT, p-mTOR/mTOR by densitometry based on immunoblot images. **(D)** Representative images of the total apoptotic cells by annexin V-FITC/PI detection. **(E)** Quantitative analysis of apoptotic cells using bar graphs. **(F)** Representative western blotting bands of LC3I, LC3II, p-AKT, AKT, p-mTOR and mTOR. **(G**, **H**, **I)** Quantitative analysis of the ratios of LC3II/LC3I, p-AKT/AKT, p-mTOR/mTOR by densitometry. Data are expressed as mean ± SD, n = 3. ^#^ *p* < 0.05, ^##^ *p* < 0.01^,###^ *p* 0.001 *versus* control group; *p < 0.05, **p < 0.01, ***p < 0.001 *versus* ISO group. ISO, isoproterenol, QLQX, Qi-Li-Qiang-Xin.

In order to investigate whether QLQX suppressed apoptosis and autophagy *via* the AKT/mTOR pathway *in vitro*, the expressions of p-AKT and p-mTOR were detected. Compared with the ISO group, QLQX and 3-MA significantly increased the levels of phosphorylated AKT and mTOR in H9c2 cells (*p* < 0.01, **Figures 7A–C**). The protein expressions of total AKT and mTOR did not show the significant difference in every group (*p* > 0.05). To further confirm whether QLQX inhibited ISO-induced apoptosis and excessive autophagy *via* activating the AKT/mTOR pathway, an AKT inhibitor capivasertib was used. As shown in **Figures 7D, E**, 50 µg/ml QLQX significantly inhibited ISO-induced apoptosis, while 3 nM capivasertib could not decrease ISO-induced cardiomyocyte apoptosis. Moreover, simultaneous administration of QLQX and capivasertib could block the effect of QLQX on inhibiting ISO-induced apoptosis. Besides, **Figures 7F–I** showed that QLQX significantly increased the ratios of p-AKT/AKT and p-mTOR/mTOR and decreased the ratio of LC3II/LC3I (*p* < 0.01), while capivasertib displayed the opposite results. All these results indicated that the AKT/mTOR signaling pathway regulated ISO-induced apoptosis and autophagy in H9c2 cells, and QLQX could inhibit ISO-induced apoptosis and autophagy-mediated cell death by activation of the AKT/mTOR pathway.

## Discussion

Isoproterenol, a well-known β-adrenoceptor agonist, can induce cardiac fibrosis and hypertrophy according to numerous published researches (Mounir et al., 2010). Fibrosis and hypertrophy often lead to cardiac remodeling and diastolic heart dysfunction (Fan et al., 2014). In accordance with previous study (Kumar et al., 2009), our result demonstrated that a subcutaneous injection of isoproterenol (5 mg/kg/d, 7 d) induced severe decline of cardiac function reflected by parameters of cardiac function, and serious cardiac fibrosis which participated in the pathogenesis of CHF observed by HE and Masson staining. Treatment by QLQX could significantly improve cardiac function confirmed by the increase of LVEF and FS. In addition, histopathological analysis revealed that QLQX treatment restored the structure of heart and significantly decreased myocardial fibrosis induced by ISO. All these data strongly showed that QLQX treatment alleviated the pathophysiological and morphologic changes induced by ISO.

It has been described that administration of ISO induced myocardial apoptosis (Hu et al., 2013). In line with the literature (Hu et al., 2013), the number of apoptotic cells significantly increased treated by ISO, while QLQX treatment showed a prominent attenuation of them. Furthermore, the related apoptosis proteins were detected in our experiment. As we know, apoptotic signaling is controlled by the Bcl-2 gene family (Shimizu et al., 1998). In terms of their role in controlling apoptosis, the Bcl-2 gene family is categorized into two subtypes: anti-apoptotic members (e.g., Bcl-2 and Bcl-xl) and pro-apoptotic members (e.g., Bax and Bak) (Rooswinkel, 2013). The balance of these two subtypes determines the activation of apoptosis (Rooswinkel, 2013). In our study, QLQX treatment decreased the level of the apoptotic Bax protein and enhanced the level of the antiapoptotic Bcl-2 protein, suggesting that QLQX has the ability to inhibit ISO-induced myocardial cell apoptosis.

The bulk of evidence has pointed out that dysfunctional myocardial autophagy is an important pathophysiological factor leading to cardiac remodeling and heart failure (Gustafsson and Gottlieb, 2008), but the role of autophagy in the progression of this condition remains controversial. In the basal state, cellular autophagy plays an antiapoptotic and cytoprotective role to preform normal function (Djordjevic, 2016). However, the consequent stressors, such as pressure overload, can result in excessive autophagy, which will enhance excessive degradation of vital cellular constituents (Hong et al., 2015; Zhang et al., 2017; Liu et al., 2018). Recent study has clearly showed that inhibiting excessive autophagy by 3-MA could alleviate myocardial injury and improve cardiac function *in vivo* and *in vitro* (Hong et al., 2015). There is a similar result observed in our present study. *In vivo*, ISO treatment induced an obvious enhance of LC3II/LC3I ratio, while MQLQX and HQLQX reduced the ratio of LC3II/LC3I, suggesting that QLQX normalized the autophagic process. *In vitro*, we also measured the another autophagic protein, P62, which is directing ubiquitinated protein to the autophagosome for degradation (Cohen-Kaplan et al., 2016). An enhanced level of P62 and a decreased level of LC3II/LC3I in QLQX group suggested that QLQX treatment inhibited the excessive activation of autophagic process *in vitro*. Importantly, these effects were similar to the changes treated by 3-MA, which could block the autophagic pathway. All these results suggested that autophagy at the end of myocardial injury would be excessively activated to overconsume proteins and organelles in cells in the model of ISO-induced myocardial injury, while QLQX treatment could normalization of autophagic processes to play the role of cardioprotection. It was noteworthy that the effect of QLQX on the regulation of autophagy was not in line with the result of Tong et al. (2018). In Tong et al.’s experiment, it suggested that the level of autophagy was activated by QLQX in the CHF rats induced by STZ. However, the level of autophagy was down-regulated by QLQX in the CHF rats induced by ISO in our study. This difference of autophagic activity could be explained by the different experimental methods (Li et al., 2016). It seemed that QLQX plays the cardioprotective role by the dual-directional regulation of autophagy to maintain the dynamic balance under different conditions of myocardial injury.

A growing number of studies clearly stated that the relation between apoptosis and autophagy is quite complex reflected by the fact that they have many common regulatory molecules, such as the PI3K/AKT/mTOR signaling pathway and Bcl-2 (Shimizu et al., 2004; Heath-Engel and Chang, 2008; Feng and Qiu, 2018). The PI3K/AKT/mTOR pathway has been confirmed that it plays a central role in the most of cellular processes that regulates growth, cell proliferation, cell survival, metabolism, and autophagy (Yu and Wei, 2016). In this pathway, PI3K activates AKT, which can, in turn, activates and phosphorylates the serine/threonine kinase mTOR *via* a cascade of regulators (Rozengurt et al., 2014). Furthermore, the activation of mTOR is considered a crucial step in autophagy inhibition (Zhu et al., 2018). Recent studies have showed that suppression of PI3K/AKT/mTOR pathway is related to inducing autophagy and apoptotic cell death *in vivo* and *in vitro* (Chen et al., 2017; Tang et al., 2017). In our present study, western blotting analysis of both *in vivo* and *in vitro* experiments revealed that ISO significantly down-regulated the expression level of p-AKT, which led to decrease of p-mTOR, shedding light on that ISO could inhibit the hyperactivation of PI3K/AKT/mTOR. However, QLQX could obviously increase p-AKT and p-mTOR expressions. All these results indicated that the potential mechanism of QLQX on alleviating ISO-induced myocardial injury was related to activating the AKT/mTOR pathway.

In conclusion, our results suggested that QLQX protected against ISO-induced injury by the inhibition of apoptosis and excessive autophagy-mediated cell death *via* activating the AKT/mTOR signaling pathway *in vivo* and *in vitro*. The interactions between QLQX and autophagy provide a new strategy to alleviate cardiomyocyte injury. However, our study still needs more deep investigations including agonists and inhibitors of autophagy to uncover more comprehensive and detailed mechanisms of the cardioprotective effect of QLQX.

## Data Availability Statement

All datasets generated for this study are included in the article/**Supplementary Material**.

## Ethics Statement

This study was carried out in accordance with the principles of the Basel Declaration and recommendations of the Statute on the Administration of Laboratory Animals, Laboratory Animal Ethics Committee of West China Hospital, Sichuan University. The protocol was approved by Laboratory Animal Ethics Committee of West China Hospital, Sichuan University.

## Author Contributions

XY, YD, and ZY contributed to the design of the experiment and supervised this work. CF and YD wrote the manuscript. GZ offered technical support in cellular experiment. CF and MY carried out *in vivo* and *in vitro* experiments and analyzed the data. XT performed the rapid identification of chemical ingredients of QLQX and determined the contents of the main components by UPLC-Q-TOF-MS. All authors have read and approved the manuscript.

## Funding

This study was supported by the National Key Research and Development Plan (2017YFC1700405) and the National Natural Science Foundation of China (81774219).

## Conflict of Interest

The authors declare that the research was conducted in the absence of any commercial or financial relationships that could be construed as a potential conflict of interest.
