# Supplementary material for: Qi-Li-Qiang-Xin Alleviates Isoproterenol-Induced Myocardial Injury by Inhibiting Excessive Autophagy via Activating AKT/mTOR Pathway
Source: Front Pharmacol. 2019 Nov 12;10:1329. doi: 10.3389/fphar.2019.01329 (PMC6861302; doi:10.3389/fphar.2019.01329)
Supplement: Supplementary file 1 [file DataSheet_1.docx]

Supplementary Material.

**Qi-Li-Qiang-Xin alleviates isoproterenol-induced myocardial injury by inhibiting excessive autophagy via activating AKT/mTOR pathway**

Legend to figures and Tables

**Fig.S1.** Characterization of 12 compounds by UPLC-Q-TOF-MS and EIC chromatograms of (1) sinapine thiocyanate, (2) calycosin-7-O-β-D-glucopyranoside, (3) hesperidin, (4) salvianolic acid B, (5) benzoylmesaconine, (6) ginsenoside Re, (7) periplocin, (8) ginsenoside Rb1, (9) formononetin, (10) periplocymarin, (11) astragalosides II and (12) alisol A in QLQX.

**Fig.S2.** The chemical structures of the main compounds of QLQX

**Fig.S3.** QLQX prevents cardiac hypertrophy and reduced ISO-induced myocardial injury *in vivo*

**Fig.S4.** Effects of QLQX on cell viability in H9c2 cells

**Table S1.** Identification of 152 compounds of QLQX by UPLC-Q-TOF-MS

**Table S2.** Content of the main compounds of QLQX


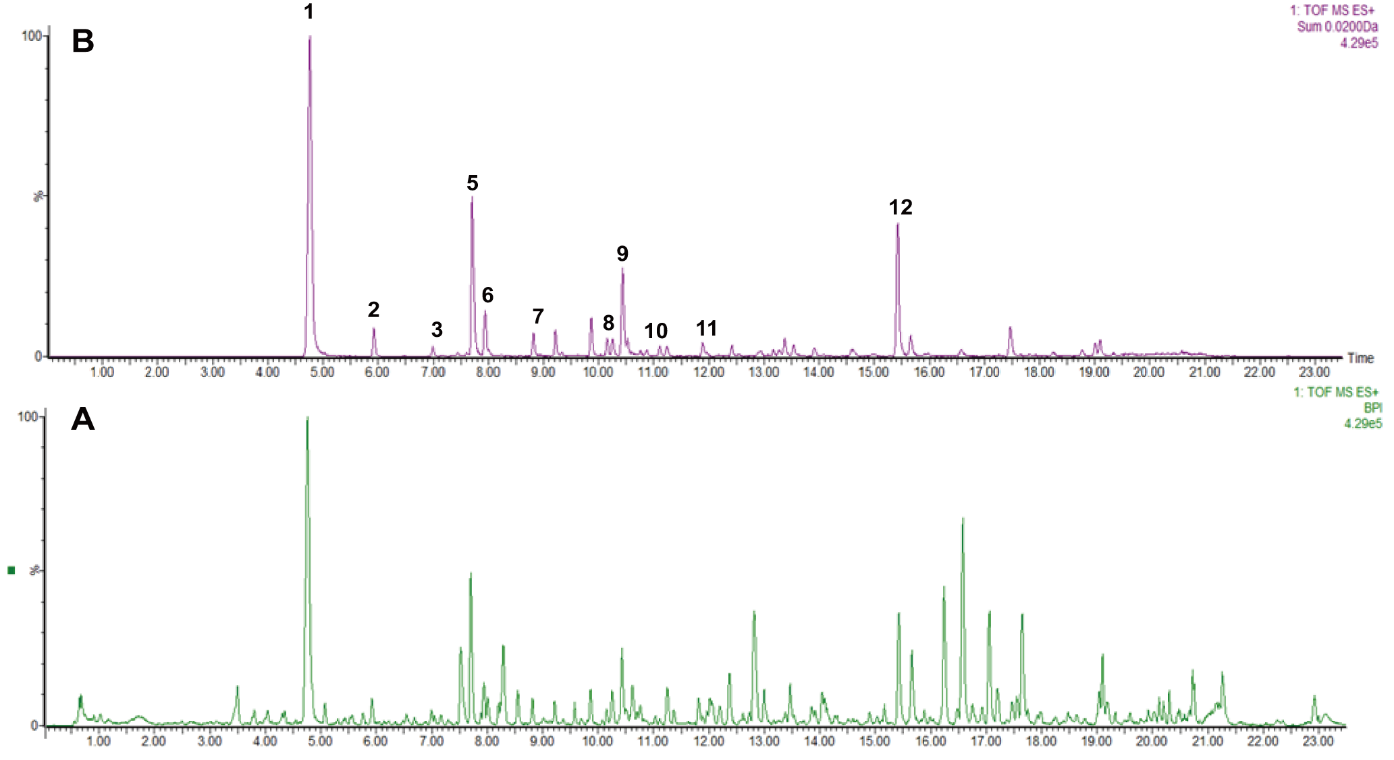


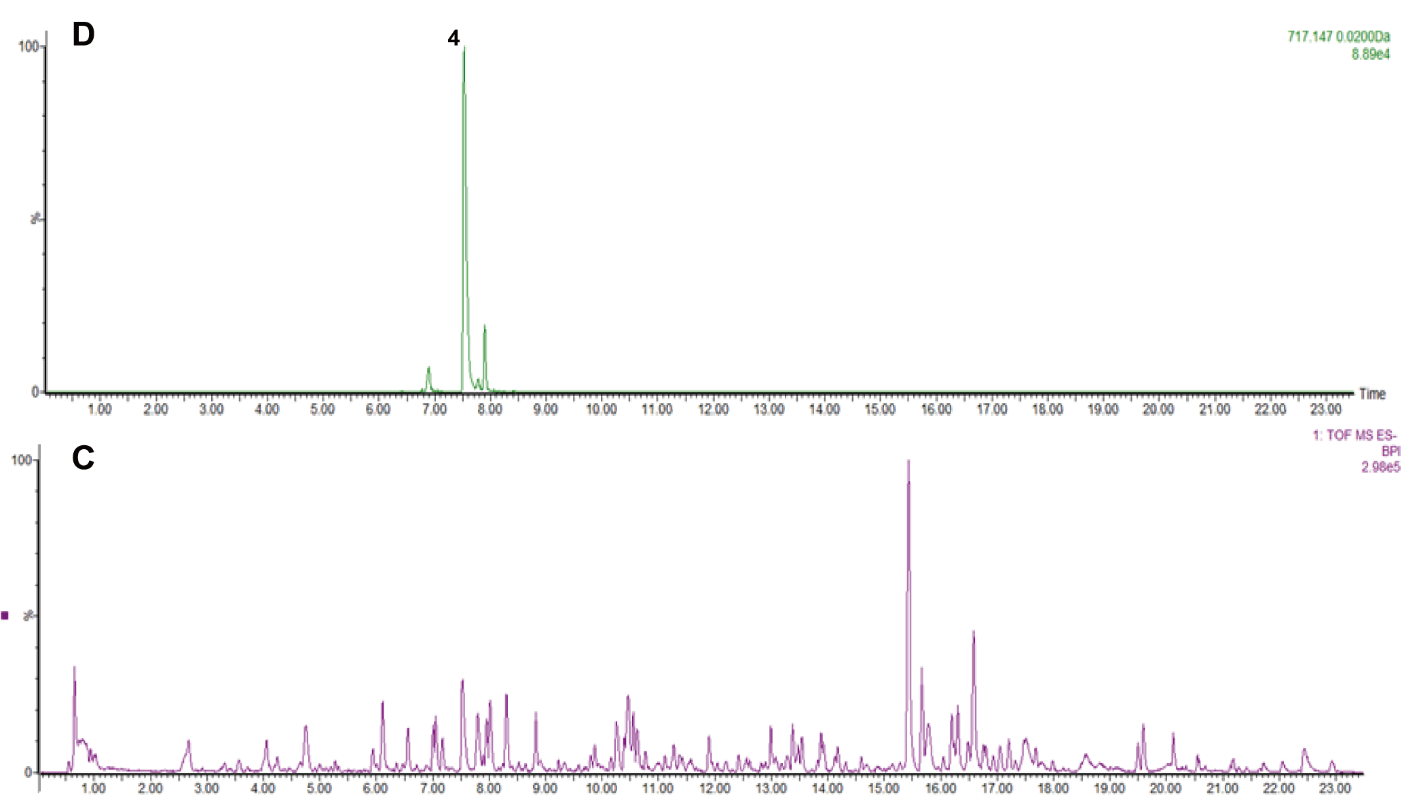


**Figure S1**. Characterization of the main compounds of QLQX by UPLC-Q-TOF-MS and EIC chromatograms of (1) sinapine thiocyanate, (2) calycosin-7-O-β-D-glucopyranoside, (3) hesperidin, (4) salvianolic acid B, (5) benzoylmesaconine, (6) ginsenoside Re, (7) periplocin, (8) ginsenoside Rb1, (9) formononetin, (10) periplocymarin, (11) astragalosides II, (12) alisol A in QLQX.

(A) QLQX in positive-ion mode (ESI^+^); (B) ESI^+^ chromatograms of the main compounds of QLQX; (C) QLQX in negative-ion mode (ESI^-^);(D) ESI^-^ chromatograms of compound **4** of QLQX.

**Condition of UPLC-Q/TOF-MS analysis:** UPLC analysis was carried out on an ACQUITYTM UPLC I-Class system comprised a quaternary pump, a diode-array detector (DAD), an autosampler, and a column compartment. Samples were separated on an Acquity UPLC BEH C18 Column (2.1 mm × 100 mm, 1.7 m) at a temperature of 40 °C. The mobile phases consisted of eluent A (0.1% formic acid in water, v/v) and eluent B (0.1% formic acid in acetonitrile, v/v) using a gradient program as follow: 0.0–1.0 min, 2% B; 1.0–18.0 min, 2–70% B; 18.0–20.0 min, 70–100% B. After holding 100% B for next 2 min, the column was returned to its starting condition. The flow rate was kept at a 0.4 mL/min and the injection volume was 2 µL for all the samples.

UPLC system was coupled to a quadrupole time-of-flight tandem mass spectrometry (SYNAPTTM G2 HDMS, Waters, Manchester, U.K.) equipped with electrospray ionization (ESI). The operating parameters were set as follow: Capillary voltage of 3 kV (ESI+) or -2.5 kV (ESI-); Sample cone voltage of 35 V; Extraction cone voltage of 4 V, source temperature of 100 °C, desolvation temperature 400 °C, cone gas flow of 50 L/h and desolvation gas flow of 800 L/h. In MSE mode, trap collision energy was 4 eV for low energy function and 20–50eV for high energy function. Argon was used as collision gas for CID in both MSE and MS2 mode. The mass spectrometer was calibrated over a range of 50-2000 Da using solution of sodium formate. Leucine-enkephalin (m/z 556.2771 in positive ion mode; m/z 554.2615 in negative ion mode) was used as external reference of LockSpray TM infused at a constant flow of 5 µL/min. Argon was used as collision gas. All data were processed with software UNIFI 1.7.0 (waters, Manchester, U.K.) and Masslynx V4.1(waters, Manchester, U.K.).


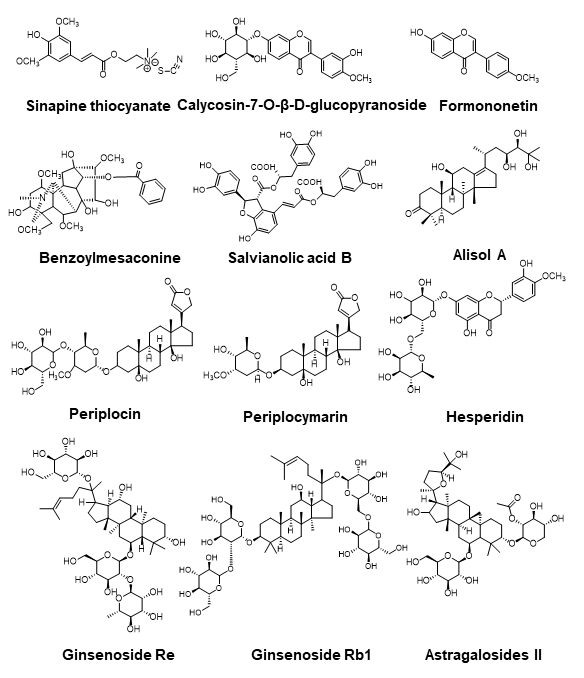


**Figure S2.** The chemical structures of the main compounds of QLQX.





**Figure S3.** QLQX prevents cardiac hypertrophy and reduced ISO-induced myocardial injury *in vivo*. Quantitative analysis of myocyte hypertrophy indices: HWI (A), LVWI (B). Quantitative analysis of myocardial injury indices by an automatic chemistry analyzer: plasma LDH level (C), plasma CK activity (D), plasma CK-MB level (E) and plasma α-HBD activity (F). Data are expressed as mean ± SD, n = 8. ^#^*p* < 0.05, ^##^*p* < 0.01, ^###^ *p* < 0.001 versus control group; ^*^*p* < 0.05, ^**^*p* < 0.01, ^***^ *p* < 0.001versus ISO group.





**Figure S4.** Effects of QLQX on cell viability in H9c2 cells. The viability of H9c2 cells was tested by MTT in different concentrations of ISO. Data are expressed as mean ± SD, n = 6. ^&^*p* < 0.05, ^&&^*p* < 0.01, ^&&&^ *p* < 0.001 versus control group.

| **Table S1. Characterization of compounds of QLQX** | | | | | | | | | |
| --- | --- | --- | --- | --- | --- | --- | --- | --- | --- |
|  | t_R_/min | selected ion | elemental composition | Measured mass | Calculated mass | Mass error | ppm | Identification | Source |
| 1 | 2.67 | [M-H]^-^ | C_9_H_9_NaO_5_ | 219.048 | 219.027 | 1.1 | 2.3 | Danshen Su | Salvia miltiorrhiza radix et rhizoma |
| 2 | 2.94 | [M+H]^+^ | C_22_H_35_NO_5_ | 394.2583 | 394.258 | -0.5 | -1.3 | Chuanfumine | Aconite lateralis radix preparata |
| 3 | 3.77 | [M+H]^+^ | C_24_H_39_NO_9_ | 486.269 | 486.268 | -0.8 | -1.6 | Mesaconine | Aconite lateralis radix preparata |
| 4 | 3.97 | [M+H]^+^ | C_22_H_35_NO_4_ | 378.2633 | 378.263 | -0.6 | -1.5 | Karakoline | Aconite lateralis radix preparata |
| 5 | 3.99 | [M+H]^+^ | C_16_H_18_O_9_ | 355.1021 | 355.103 | -0.8 | -2.3 | Chlorogenic acid | Cortex periplocae |
| 6 | 4.05 | [M+H]^+^ | C_23_H_37_NO_5_ | 408.274 | 408.274 | -0.5 | -1.2 | Isotalatizidine | Aconite lateralis radix preparata |
| 7 | 4.23 | [M-H]^-^ | C_27_H_30_O_17_ | 625.141 | 625.142 | 0.9 | 0.6 | Quercetin-7-*O*-β-D-gentiobioside | Semen descurainiae |
| 8 | 4.23 | [M+H]^+^ | C_22_H_31_NO_3_ | 358.238 | 358.238 | 0.3 | 0.9 | Songorine | Aconite lateralis radix preparata |
| 9 | 4.26 | [M-H]^-^ | C_33_H_40_O_21_ | 771.201 | 771.203 | 2.1 | 2.7 | Kaempferol­-3­-*O*­-β-­D­glucopyranosyl­7-­*O*­-β­gentiobioside | Semen descurainiae |
| 10 | 4.38 | [M-H]^-^ | C_34_H_42_O_22_ | 801.2082 | 801.207 | -1.3 | -1.6 | Isorhamnetin­-3­-*O-*­β-­D­glucopyranosyl-­7­-*O*­-β­gentiobioside | Semen descurainiae |
| 11 | 4.64 | [M-SCN]^+^ | C_16_H_24_NO_5_ | 310.1649 | 310.164 | -1.4 | -0.4 | Sinapine thiocyanate* | Semen descurainiae |
| 12 | 4.78 | [M-H]^-^ | C_28_H_32_O_17_ | 639.1573 | 639.158 | 0.6 | 1.0 | Isorhamnetin­-3,7-­di­-*O-*β-­D-­glucopyranosyl | Semen descurainiae |
| 13 | 4.78 | [M+H]^+^ | C_24_H_39_NO_8_ | 470.274 | 470.273 | -0.9 | -1.8 | Hypaconine | Aconite lateralis radix preparata |
| 14 | 4.82 | [M+H]^+^ | C_24_H_39_NO_7_ | 454.2795 | 454.279 | -0.4 | -1.0 | Fuziline | Aconite lateralis radix preparata |
| 15 | 5.02 | [M-H]^-^ | C_23_H_32_O_15_ | 547.1668 | 547.167 | 0.3 | 0.6 | 1-sinapoylgentiobiose | Semen descurainiae |
| 16 | 5.05 | [M+H]^+^ | C_24_H_39_NO_6_ | 438.2855 | 438.286 | 0.5 | 1.1 | Neoline | Aconite lateralis radix preparata |
| 17 | 5.19 | [M+H]^+^ | C_33_H_42_O_19_ | 743.24 | 741.24 | 0.1 | 0.1 | Naringin 4'-*O*-glucoside | Citri reticulatae pericarpium |
| 18 | 5.24 | [M+H]^+^ | C_24_H_39_NO_6_ | 438.2842 | 438.284 | -0.8 | -1.9 | Foresticine | Aconite lateralis radix preparata |
| 19 | 5.49 | [M+H]^+^ | C_24_H_39_NO_5_ | 422.2889 | 422.288 | -1.2 | -2.9 | Talatizamine | Aconite lateralis radix preparata |
| 20 | 5.61 | [M-H]^-^ | C_27_H_30_O_16_ | 609.147 | 609.148 | 0.9 | 1.5 | Rutin | Carthami flos |
| 21 | 5.79 | [M+H]^+^ | C_10_H_8_O_4_ | 193.0502 | 193.051 | 0.7 | 3.4 | Scopoletin | Cortex periplocae |
| 22 | 5.94 | [M+H]^+^ | C_22_H_22_O_10_ | 447.1287 | 447.129 | 0.1 | 0.2 | Calycosin-7-*O*-β-D-glucopyranoside* | Aastragali radix |
| 23 | 6.01 | [M-H]^-^ | C_21_H_20_O_12_ | 463.0858 | 463.083 | -2.4 | -2.2 | Quercetin-­3-­*O-*­β­-D­glucopyranoside | Semen descurainiae |
| 24 | 6.07 | [M+H]^+^ | C_25_H_41_NO_6_ | 452.2999 | 452.299 | -0.7 | -1.6 | Chasmanine | Aconite lateralis radix preparata |
| 25 | 6.27 | [M-H]^-^ | C_18_H_19_NO_5_ | 328.119 | 328.118 | -1.2 | -3.6 | N-*trans*-feruloyloctopamine | Polygonati odorati rhizoma |
| 26 | 6.36 | [M-H]^-^ | C_27_H_30_O_15_ | 593.1542 | 593.151 | 3.0 | 5.0 | Kaempferol-3-*O*- β-rutinoside | Carthami flos |
| 27 | 6.48 | [M+H]^+^ | C_26_H_41_NO_6_ | 464.3008 | 464.301 | 0.2 | 0.4 | 14-Acetyltalatizamine | Aconite lateralis radix preparata |
| 28 | 6.56 | [M+H]^+^ | C_27_H_32_O_14_ | 581.1879 | 581.187 | -1.1 | -1.8 | Naringin | Citri reticulatae pericarpium |
| 29 | 6.62 | [M+H]^+^ | C_31_H_43_NO_11_ | 606.2919 | 606.293 | 1.0 | 1.7 | 14-Benzoyl-10-OH-mesaconine | Aconite lateralis radix preparata |
| 30 | 6.65 | [M-H]^-^ | C_26_H_28_O_14_ | 563.1398 | 563.139 | -0.9 | -1.6 | Drabanemoroside | Semen descurainiae |
| 31 | 6.72 | [M-H]^-^ | C_22_H_22_O_12_ | 477.1024 | 477.101 | -1.5 | -3.1 | Isorhamnetin­3­-*O-*­β-­D­glucopyranoside | Semen descurainiae |
| 32 | 6.98 | [M+H]^+^ | C_28_H_34_O_15_ | 611.1964 | 611.196 | -0.7 | -1.9 | Hesperidin* | Citri reticulatae pericarpium |
| 33 | 6.99 | [M-H]^-^ | C_18_H_16_O_8_ | 359.0772 | 359.077 | 0.0 | 0.0 | Rosmarinic acid | Salvia miltiorrhiza radix et rhizoma |
| 34 | 7 | [M-H]^-^ | C_36_H_30_O_16_ | 717.1472 | 717.146 | -1.1 | -1.8 | Salvianolic acid E | Salvia miltiorrhiza radix et rhizoma |
| 35 | 7.09 | [M+H]^+^ | C_9_H_6_O_2_ | 147.045 | 147.045 | 0.4 | 2.7 | Coumarin | Cinnamomi ramulus |
| 36 | 7.11 | [M-H]^-^ | C_27_H_22_O_12_ | 537.1001 | 537.096 | -3.7 | -7.0 | Lithospermic acid A | Salvia miltiorrhiza radix et rhizoma |
| 37 | 7.15 | [M-H]^-^ | C_34_H_42_O_19_ | 753.2247 | 753.225 | -0.2 | -0.3 | 1,2-disinapoylgentiobiose | Semen descurainiae |
| 38 | 7.44 | [M-H]^-^ | C_36_H_30_O_16_ | 717.1474 | 717.146 | -1.3 | -1.8 | Salvianolic acid B* | Salvia miltiorrhiza radix et rhizoma |
| 39 | 7.62 | [M+Na]^+^ | C_47_H_80_O_18_ | 955.5218 | 955.52 | -1.9 | -2.0 | Notoginsenoside R1 | Ginseng radix et rhizoma |
| 40 | 7.62 | [M+H]^+^ | C_31_H_43_NO_10_ | 590.2961 | 590.296 | 0.2 | 0.3 | Benzoylmesaconine* | Aconite lateralis radix preparata |
| 41 | 7.75 | [M-H]^-^ | C_28_H_32_O_14_ | 591.1719 | 591.171 | -0.9 | -1.6 | 1,2-di-O-sinapoyl-β-D-glucopyranose | Semen descurainiae |
| 42 | 7.76 | [M+H]^+^ | C_22_H_22_O_9_ | 431.1324 | 431.131 | -1.2 | -2.9 | Ononin | Aastragali radix |
| 43 | 7.85 | [M-H]^-^ | C_36_H_30_O_16_ | 717.1472 | 717.146 | -1.1 | -1.8 | Isosalvianolic acid B | Salvia miltiorrhiza radix et rhizoma |
| 44 | 7.94 | [M+Na]^+^ | C_48_H_82_O_18_ | 969.5385 | 969.538 | -0.8 | -0.9 | Ginsenoside Re* | Ginseng radix et rhizoma |
| 45 | 7.94 | [M+Na]^+^ | C_42_H_72_O_14_ | 823.4813 | 823.481 | -0.1 | -0.1 | Ginsenoside Rg1 | Ginseng radix et rhizoma |
| 46 | 7.99 | [M-H]^-^ | C_26_H_22_O_10_ | 493.1143 | 493.113 | -0.6 | -1.3 | Salvianolic acid A | Salvia miltiorrhiza radix et rhizoma |
| 47 | 8.15 | [M+H]^+^ | C_32_H_45_NO_10_ | 604.3127 | 604.314 | 1.0 | 1.7 | Benzoylaconine | Aconite lateralis radix preparata |
| 48 | 8.3 | [M+H]^+^ | C_16_H_12_O_5_ | 285.0763 | 285.077 | 0.5 | 1.9 | Calycosin | Aastragali radix |
| 49 | 8.44 | [M+Na]^+^ | C_33_H_54_O_12_ | 665.3499 | 665.349 | -0.9 | -1.3 | periseoside C | Cortex periplocae |
| 50 | 8.49 | [M+H]^+^ | C_31_H_43_NO_9_ | 574.301 | 574.301 | -0.1 | -0.1 | Benzoylhypaconine | Aconite lateralis radix preparata |
| 51 | 8.51 | [M+H]^+^ | C_28_H_34_O_14_ | 595.202 | 595.202 | -0.7 | -1.2 | Poncirin | Citri reticulatae pericarpium |
| 52 | 8.53 | [M+H]^+^ | C_9_H_8_O_2_ | 149.0609 | 149.06 | 0.4 | 4.0 | cinnamic acid | Cinnamomi ramulus |
| 53 | 8.54 | [M+H]^+^ | C_23_H_28_O_10_ | 465.1736 | 465.176 | -2.5 | -5.5 | Isomucronulatol-7-*O*-β-D-glucoside | Aastragali radix |
| 54 | 8.61 | [M+Na]^+^ | C_29_H_42_O_9_ | 557.2726 | 557.273 | 0.5 | 0.9 | helveticoside | Semen descurainiae |
| 55 | 8.81 | [M+Na]^+^ | C_36_H_56_O_13_ | 719.3585 | 719.356 | -2.5 | -3.5 | periplocin* | Cortex periplocae |
| 56 | 8.89 | [M+H]^+^ | C_31_H_43_NO_8_ | 558.3063 | 558.307 | 0.2 | 0.4 | 14-benzoyl-13-deoxyhyaconine | Aconite lateralis radix preparata |
| 57 | 8.93 | [M+H]^+^ | C_33_H_45_NO_12_ | 648.2998 | 648.298 | -1.7 | -2.6 | Beiwutine | Aconite lateralis radix preparata |
| 58 | 8.97 | [M+H]^+^ | C_9_H_8_O_1_ | 133.0659 | 133.065 | 0.6 | 4.5 | cinnamaldehyde | Cinnamomi ramulus |
| 59 | 8.98 | [M-H]^-^ | C_34_H_37_N_3_O_6_ | 582.2595 | 582.26 | -0.9 | -1.5 | N1,N5-(Z)–N10-(E)-tri-p-coumaroylspermidine | Carthami flos |
| 60 | 8.99 | [M+H]^+^ | C_32_H_45_NO_9_ | 588.3167 | 588.314 | -3.2 | -1.9 | Benzoyldeoxyaconine | Aconite lateralis radix preparata |
| 61 | 9.23 | [M+Na]^+^ | C_23_H_34_O_5_ | 413.2296 | 413.229 | -0.2 | -0.5 | periplogenin | Cortex periplocae |
| 62 | 9.39 | [M+H]^+^ | C_27_H_32_O_14_ | 581.1866 | 581.187 | -0.6 | -0.7 | Narirutin isomer | Citri reticulatae pericarpium |
| 63 | 9.54 | [M-H]- | C_16_H_12_O_7_ | 315.051 | 315.051 | -0.1 | -0.2 | isorhamnetin | Semen descurainiae |
| 64 | 9.62 | [M+H]^+^ | C_33_H_45_NO_11_ | 632.3065 | 632.307 | 0.8 | 0.5 | Mesaconitine | Aconite lateralis radix preparata |
| 65 | 9.68 | [M+Na]^+^ | C_54_H_90_O_24_ | 1145.5714 | 1145.57 | -2.2 | -2.6 | Plocoside B | Cortex periplocae |
| 66 | 9.73 | [M+Na]^+^ | C_33_H_56_O_17_ | 747.341 | 747.34 | -1.1 | -1.4 | Oligosaccharide F_2_ | Cortex periplocae |
| 67 | 9.82 | [M+Na]^+^ | C_40_H_66_O_16_ | 825.4273 | 825.43 | 3.0 | 3.6 | Glycoside K | Cortex periplocae |
| 68 | 9.88 | [M+Na]^+^ | C_42_H_72_O_14_ | 823.4808 | 823.48 | -0.7 | -0.8 | Ginsenoside Rf | Ginseng radix et rhizoma |
| 69 | 10.16 | [M+Na]^+^ | C_58_H_98_O_26_ | 1233.623 | 1233.62 | 0.8 | 0.7 | Ginsenoside Ra1 | Ginseng radix et rhizoma |
| 70 | 10.17 | [M+Na]^+^ | C_41_H_70_O_13_ | 793.4704 | 793.471 | 0.5 | 0.6 | Ginsenoside F5 | Ginseng radix et rhizoma |
| 71 | 10.25 | [M+Na]^+^ | C_54_H_92_O_23_ | 1131.592 | 1131.59 | -0.2 | -0.2 | Ginsenoside Rb1* | Ginseng radix et rhizoma |
| 72 | 10.29 | [M+H]^+^ | C_33_H_45_NO_10_ | 616.3121 | 616.313 | 0.4 | 0.7 | Hypaconitine | Aconite lateralis radix preparata |
| 73 | 10.46 | [M+Na]^+^ | C_42_H_72_O_13_ | 807.4865 | 807.487 | 0.0 | 0.0 | 20(S)-Ginsenoside Rg2 | Ginseng radix et rhizoma |
| 74 | 10.46 | [M+H]^+^ | C_16_H_12_O_4_ | 269.0804 | 269.08 | -0.5 | -1.7 | Formononetin* | Aastragali radix |
| 75 | 10.49 | [M+H]^+^ | C_58_H_98_O_26_ | 1211.6378 | 1211.63 | -4.2 | -3.4 | Ginsenoside Ra2 | Ginseng radix et rhizoma |
| 76 | 10.52 | [M+Na]^+^ | C_53_H_90_O_22_ | 1101.5803 | 1101.58 | -1.3 | -1.2 | Ginsenoside Rc | Ginseng radix et rhizoma |
| 77 | 10.54 | [M+Na]^+^ | C_36_H_62_O_9_ | 661.4267 | 661.429 | 1.9 | 3.0 | 20(R)-Ginsenoside Rh1 | Ginseng radix et rhizoma |
| 78 | 10.59 | [M+Na]^+^ | C_27_H_44_O_6_ | 487.3021 | 487.301 | -0.9 | -1.9 | Periplocoside N | Cortex periplocae |
| 79 | 10.61 | [M+Na]^+^ | C_48_H_76_O_19_ | 979.4857 | 979.484 | -1.6 | -1.6 | Ginsenoside Ro | Ginseng radix et rhizoma |
| 80 | 10.66 | [M+H]^+^ | C_47_H_78_O_19_ | 947.5191 | 947.521 | 1.9 | 2.1 | AstragalosidesV/VI/VII | Aastragali radix |
| 81 | 10.74 | [M+H]^+^ | C_17_H_16_O_5_ | 301.107 | 301.105 | -2.0 | -0.6 | (6a*R*,11a*R*)-9,10-dimethoxy-pterocarpan | Aastragali radix |
| 82 | 10.77 | [M+Na]^+^ | C_53_H_90_O_22_ | 1101.5804 | 1101.58 | -1.2 | -1.1 | Ginsenoside Rb2 | Ginseng radix et rhizoma |
| 83 | 10.79 | [M+Na]^+^ | C_35_H_60_O_17_ | 775.3727 | 775.374 | 0.1 | 0.0 | Perisesaccharide C | Cortex periplocae |
| 84 | 10.89 | [M+Na]^+^ | C_30_H_46_O_8_ | 557.3085 | 557.309 | 0.0 | 0.0 | Periplocymarin | Cortex periplocae |
| 85 | 11.02 | [M+Na]^+^ | C_56_H_94_O_24_ | 1173.5995 | 1173.6 | 2.5 | 2.6 | Quinquenoside R1 | Ginseng radix et rhizoma |
| 86 | 11.05 | [M+H]^+^ | C_30_H_48_O_6_ | 505.3532 | 505.354 | 0.9 | 1.7 | 16-oxo-Alisol A | Alismatis rhizoma |
| 87 | 11.12 | [M+H]^+^ | C_41_H_68_O_14_ | 785.467 | 785.469 | -1.7 | -2.2 | Astragalosides IV | Aastragali radix |
| 88 | 11.23 | [M+Na]^+^ | C_41_H_68_O_14_ | 807.4504 | 807.451 | 0.2 | 0.3 | Isoastragalosides IV | Aastragali radix |
| 89 | 11.28 | [M+Na]^+^ | C_35_H_58_O_18_ | 789.3491 | 789.352 | -3.0 | -3.8 | Perisesaccharide E | Cortex periplocae |
| 90 | 11.36 | [M-H]^-^ | C_17_H_16_O_5_ | 299.0925 | 299.092 | -0.6 | -2.5 | 5,7,4'-Trihydroxy-6-methyl homoisoflavanone | Polygonati odorati rhizoma |
| 91 | 11.38 | [M+Na]^+^ | C_48_H_82_O_18_ | 969.5362 | 969.533 | -3.2 | -3.3 | Ginsenoside Rd | Ginseng radix et rhizoma |
| 92 | 11.41 | [M+Na]^+^ | C_32_H_50_O_7_ | 569.3445 | 569.344 | -0.4 | -0.7 | 16-oxo-alisol A 23-actetate | Alismatis rhizoma |
| 93 | 11.65 | [M-H]^-^ | C_18_H_18_O_6_ | 329.1025 | 329.102 | -0.6 | -1.8 | 5,7,4′-Trihydroxy-6-methyl-8-methoxyl homoisoflavanone | Polygonati odorati rhizoma |
| 94 | 11.82 | [M+H]^+^ | C_21_H_22_O_8_ | 403.1388 | 403.139 | 0.0 | 0.0 | Nobiletin | Citri reticulatae pericarpium |
| 95 | 11.9 | [M+H]^+^ | C_43_H_70_O_15_ | 827.4754 | 827.479 | -2.1 | -2.5 | Astragalosides II* | Aastragali radix |
| 96 | 11.97 | [M+H]^+^ | C_32_H_50_O_7_ | 547.3622 | 547.362 | -0.7 | -1.3 | 16-oxo-alisol A 24-actetate | Alismatis rhizoma |
| 97 | 12.02 | [M-H]^-^ | C_18_H_18_O_5_ | 313.1081 | 313.108 | -0.1 | -0.4 | 5,7,4'-Trihydroxyl-6,8-dimethyl homoisoflavanone | Polygonati odorati rhizoma |
| 98 | 12.06 | [M+Na]^+^ | C_56_H_92_O_25_ | 1187.5794 | 1187.58 | -2.6 | -2.2 | Glycoside H2/ isomer | Cortex periplocae |
| 99 | 12.2 | [M+H]^+^ | C_48_H_78_O_18_ | 943.5238 | 943.526 | 2.3 | 2.4 | Soyasaponin I | Aastragali radix |
| 100 | 12.38 | [M+H]^+^ | C_22_H_24_O_9_ | 433.1494 | 433.15 | 0.1 | 0.1 | 3,5,6,7,8,3',4'-heptamethoxyflavone | Citri reticulatae pericarpium |
| 101 | 12.39 | [M+H]^+^ | C_43_H_70_O_15_ | 827.4778 | 827.479 | 0.9 | 1.2 | Isoastragalosides II | Aastragali radix |
| 102 | 12.56 | [M+Na]^+^ | C_30_H_50_O_6_ | 529.3498 | 529.35 | -0.1 | -0.2 | 13,17-epoxy-alisol A | Alismatis rhizoma |
| 103 | 12.63 | [M+H]^+^ | C_56_H_92_O_25_ | 1165.597 | 1165.6 | -3.0 | -2.6 | Glycoside H2/ isomer | Cortex periplocae |
| 104 | 12.68 | [M+H]^+^ | C_21_H_22_O_9_ | 419.1333 | 419.134 | -0.9 | -2.1 | Natsudaidain | Citri reticulatae pericarpium |
| 105 | 12.76 | [M+H]^+^ | C_20_H_20_O_7_ | 373.1274 | 373.127 | -0.8 | -2.1 | Tangeretin | Citri reticulatae pericarpium |
| 106 | 13.02 | [M+H]^+^ | C_30_H_46_O_5_ | 487.341 | 487.34 | -0.8 | -1.7 | Alisol C | Alismatis rhizoma |
| 107 | 13.03 | [M+Na]^+^ | C_56_H_92_O_24_ | 1171.5864 | 1171.59 | 0.6 | 0.7 | Glycoside H1 | Cortex periplocae |
| 108 | 13.07 | [M+H]^+^ | C_30_H_48_O_5_ | 489.3555 | 489.354 | -1.9 | -3.9 | 16-oxo-11-deoxy-Alisol A | Alismatis rhizoma |
| 109 | 13.19 | [M+Na]^+^ | C_42_H_72_O_12_ | 789.4751 | 789.477 | -1.4 | -1.8 | Ginsenoside Rg6/Rg4 | Ginseng radix et rhizoma |
| 110 | 13.29 | [M+Na]^+^ | C_36_H_60_O_8_ | 643.4156 | 643.419 | -3.0 | -4.7 | Ginsenoside Rh4/Rk3 | Ginseng radix et rhizoma |
| 111 | 13.39 | [M+Na]^+^ | C_45_H_72_O_16_ | 891.4699 | 891.469 | -1.3 | -1.5 | Astragalosides I | Aastragali radix |
| 112 | 13.48 | [M+Na]^+^ | C_19_H_22_O_4_ | 337.1406 | 337.141 | 0.4 | 1.4 | Neocryptotanshinone | Salvia miltiorrhiza radix et rhizoma |
| 113 | 13.52 | [M+Na]^+^ | C_32_H_48_O_6_ | 551.3353 | 551.336 | 0.9 | 1.7 | Alisol C 23-acetate isomer | Alismatis rhizoma |
| 114 | 13.75 | [M+Na]^+^ | C_32_H_52_O_7_ | 571.3602 | 571.36 | -0.3 | -0.6 | 13,17-epoxy-Alisol A 24-acetate | Alismatis rhizoma |
| 115 | 13.75 | [M+Na]^+^ | C_32_H_50_O_6_ | 553.35 | 553.35 | -0.1 | -0.3 | Alisol N 23-acetate | Alismatis rhizoma |
| 116 | 13.75 | [M+Na]^+^ | C_15_H_24_O_2_ | 259.1668 | 259.167 | 0.3 | 1.0 | Orientalol C | Alismatis rhizoma |
| 117 | 13.89 | [M+Na]^+^ | C_18_H_14_O_3_ | 301.0838 | 301.084 | 0.3 | 0.9 | 15,16-Dihydrotanshinone I | Salvia miltiorrhiza radix et rhizoma |
| 118 | 13.91 | [M+Na]^+^ | C_45_H_72_O_16_ | 891.4709 | 891.471 | -0.3 | -0.4 | Isoastragalosides I | Aastragali radix |
| 119 | 14.07 | [M+Na]^+^ | C_32_H_48_O_6_ | 551.3336 | 551.333 | -0.7 | -1.3 | Alisol C 23-acetate | Alismatis rhizoma |
| 120 | 14.09 | [M+Na]^+^ | C_30_H_48_O_5_ | 511.3391 | 511.339 | 0.2 | 0.5 | Alisol F | Alismatis rhizoma |
| 121 | 14.1 | [M+Na]^+^ | C_42_H_72_O_13_ | 807.4835 | 807.487 | 3.0 | 3.7 | 20(S)-Ginsenoside F_2_ | Ginseng radix et rhizoma |
| 122 | 14.17 | [M+H]^+^ | C_32_H_48_O_6_ | 529.351 | 529.353 | -0.4 | -0.7 | Alisol C 23-acetate | Alismatis rhizoma |
| 123 | 14.29 | [M+Na]^+^ | C_42_H_72_O_13_ | 807.4835 | 807.487 | 3.0 | 3.7 | *20(S)-*Ginsenoside Rg3 | Ginseng radix et rhizoma |
| 124 | 14.29 | [M+Na]^+^ | C_30_H_44_O_4_ | 491.3086 | 491.312 | 3.2 | 3.5 | Alisol L | Alismatis rhizoma |
| 125 | 14.55 | [M+H]^+^ | C_30_H_46_O_5_ | 487.3407 | 487.342 | -1.6 | -2.3 | 16-oxo-11-anhydro-Alisol A | Alismatis rhizoma |
| 126 | 14.61 | [M+Na]^+^ | C_45_H_72_O_16_ | 891.472 | 891.473 | 0.8 | 0.9 | Astragalosides I isomer | Aastragali radix |
| 127 | 14.67 | [M+Na]^+^ | C_30_H_50_O_5_ | 513.355 | 513.355 | 0.0 | 0.2 | Alisol E | Alismatis rhizoma |
| 128 | 14.7 | [M+Na]^+^ | C_19_H_22_O_4_ | 337.1411 | 337.141 | 0.1 | 0.3 | Tanshinone V | Salvia miltiorrhiza radix et rhizoma |
| 129 | 14.93 | [M+H]^+^ | C_20_H_30_O_2_ | 303.2325 | 303.23 | 0.1 | 0.3 | 16(R)-entkaurane-2,12-dione | Alismatis rhizoma |
| 130 | 15.07 | [M+Na]^+^ | C_32_H_50_O_6_ | 553.3494 | 553.349 | -0.6 | -1.1 | Alisol F 24-acetate | Alismatis rhizoma |
| 131 | 15.42 | [M+H]^+^ | C_30_H_50_O_5_ | 491.3735 | 491.374 | 0.4 | 0.9 | Alisol A* | Alismatis rhizoma |
| 132 | 15.66 | [M+Na]^+^ | C_19_H_20_O_3_ | 319.13 | 319.13 | -0.5 | -1.4 | Cryptotanshione | Salvia miltiorrhiza radix et rhizoma |
| 133 | 15.69 | [M+Na]^+^ | C_32_H_52_O_6_ | 555.3662 | 555.367 | 0.6 | 1.1 | Alisol A 23-acetate | Alismatis rhizoma |
| 134 | 15.88 | [M+Na]^+^ | C_43_H_70_O_13_ | 817.4707 | 817.471 | -0.7 | -0.9 | Perisepiumoside E | Cortex periplocae |
| 135 | 16.03 | [M+H]^+^ | C_60_H_100_O_23_ | 1201.671 | 1201.67 | -2.0 | -1.7 | Periplocoside J | Cortex periplocae |
| 136 | 16.47 | [M+Na]^+^ | C_42_H_70_O_12_ | 789.4752 | 789.476 | 0.7 | 1.0 | Ginsenoside Rg5 | Ginseng radix et rhizoma |
| 137 | 16.61 | [M+Na]^+^ | C_32_H_52_O_6_ | 555.3657 | 555.366 | 0.0 | 0.1 | Alisol A 24-acetate | Alismatis rhizoma |
| 138 | 16.93 | [M+Na]^+^ | C_32_H_48_O_5_ | 535.3386 | 535.338 | -0.8 | -1.5 | 11-Deoxy-alisol C 23-acetate | Alismatis rhizoma |
| 139 | 17.49 | [M+Na]^+^ | C_30_H_48_O_4_ | 495.3435 | 495.343 | -0.9 | -1.9 | Alisol G | Alismatis rhizoma |
| 140 | 17.51 | [M+Na]^+^ | C_19_H_18_O_3_ | 317.1141 | 317.113 | -0.7 | -2.2 | Tanshinone IIA | Salvia miltiorrhiza radix et rhizoma |
| 141 | 17.55 | [M+Na]^+^ | C_63_H_104_O_23_ | 1251.6847 | 1251.68 | -1.4 | -1.1 | Periplocoside F | Cortex periplocae |
| 142 | 17.66 | [M+Na]^+^ | C_30_H_44_O_4_ | 491.3086 | 491.312 | 3.2 | 3.5 | Alisol L isomer | Alismatis rhizoma |
| 143 | 17.76 | [M+Na]^+^ | C_30_H_46_O_4_ | 493.328 | 493.327 | -0.9 | -1.8 | 16,23-Oxido-alisol B | Alismatis rhizoma |
| 144 | 17.82 | [M+Na]^+^ | C_30_H_48_O_4_ | 495.3435 | 495.343 | -0.9 | -1.9 | Alisol B | Alismatis rhizoma |
| 145 | 17.99 | [M+Na]^+^ | C_30_H_50_O_4_ | 497.3592 | 497.36 | 0.9 | 1.5 | 11-deoxy-alisol A | Alismatis rhizoma |
| 146 | 18.28 | [M+Na]^+^ | C_32_H_50_O_5_ | 537.356 | 537.355 | 0.5 | 0.8 | Alisol B 23-acetate isomer | Alismatis rhizoma |
| 147 | 18.51 | [M+Na]^+^ | C_32_H_52_O_5_ | 539.373 | 539.371 | 1.8 | 3.3 | 25-Dehydroxy-24-acetate alisol A isomer | Alismatis rhizoma |
| 148 | 19.11 | [M+Na]^+^ | C_32_H_50_O_5_ | 537.3552 | 537.355 | 0.2 | 0.3 | Alisol B 23-acetate | Alismatis rhizoma |
| 149 | 19.52 | [M+Na]^+^ | C_70_H_110_O_27_ | 1405.7091 | 1405.91 | -3.5 | -2.5 | Periperoxide A | Cortex periplocae |
| 150 | 19.67 | [M+Na]^+^ | C_70_H_112_O_26_ | 1391.7262 | 1391.72 | -7.2 | -5.5 | Periplocoside D | Cortex periplocae |
| 151 | 20.19 | [M+Na]^+^ | C_72_H_114_O_27_ | 1433.7447 | 1433.75 | 0.8 | 0.5 | Periplocoside A | Cortex periplocae |
| 152 | 20.46 | [M+Na]^+^ | C_32_H_50_O_4_ | 521.3602 | 521.36 | 0.1 | 0.2 | 11-Deoxy-alisol B 23-acetate | Alismatis rhizoma |

| **Table S2**. Contents of the main compounds of QLQX | | |
| --- | --- | --- |
| NO.1 | Compound | Concentration (mg/g) |
| 1 | Sinapine thiocyanate | 0.79 |
| 2 | Calycosin-7-O-β-D-glucopyranoside | 0.25 |
| 3 | Hesperidin | 0.20 |
| 4 | Salvianolic acid B | 3.13 |
| 5 | Benzoylmesaconine | 0.10 |
| 6 | Ginsenoside Re | 0.43 |
| 7 | Periplocin | 0.31 |
| 8 | Ginsenoside Rb1 | 1.15 |
| 9 | Formononetin | 0.08 |
| 10 | Periplocymarin | 0.27 |
| 11 | Astragalosides II | 0.51 |
| 12 | Alisol A | 0.62 |
| Contents of the main compounds of QLQX was measured by the external standard method. | | |
